# An Enhanced Black-Winged Kite Algorithm with Multiple Strategies for Global Optimization and Constrained Engineering Applications

**DOI:** 10.3390/biomimetics11050309

**Published:** 2026-05-01

**Authors:** Chengtao Du, Jinzhong Zhang, Jie Fang

**Affiliations:** School of Electrical and Photoelectronic Engineering, West Anhui University, Lu’an 237012, China; ctdu@wxc.edu.cn (C.D.); fangjie@wxc.edu.cn (J.F.)

**Keywords:** black-winged kite algorithm, ranking-based differential mutation, simplex method, elite opposition-based learning strategy, benchmark functions, engineering designs

## Abstract

The black-winged kite algorithm (BKA) integrates the Cauchy mutation strategy and the leader selection strategy to simulate high-altitude circling exploration, fixed-point diving attack, and group cooperative migration of the black-winged kites to approximate the global optimal solution. The BKA exhibits deficiencies in ponderous convergence efficacy, inefficient calculation precision, and insufficient population diversity. To strengthen the convergence property and computational practicability, an enhanced BKA with multiple strategies (MSBKA) is advocated to accommodate global optimization and constrained engineering applications. The objective is to systematically verify its advancement and competitiveness and accurately actualize the global optimal solution. The ranking-based differential mutation can strengthen population information interaction, accelerate convergence efficiency, restrain premature convergence, diminish homogenization competition, promote exploration and exploitation, intensify elite individual guidance, downscale ineffective iterations, and materialize orderly population renewal. The simplex method can execute the local refinement operations of reflection, expansion, compression and contraction, strengthen local mining efficiency, ameliorate solution accuracy, abate parameter sensitivity, eschew local optimal traps, accelerate accurate convergence, and preserve the optimal individual potential. The elite opposition-based learning strategy can fabricate reverse solutions, expand the monolithic detection space, shorten the convergence process, elevate the quality of initial and iterative solutions, boost population diversity, guide intelligent search direction, and relieve premature convergence. The MSBKA utilizes deficiency orientation, strategy adaptation, and collaborative search to accomplish the realistic demands of high-precision, high-efficiency and strong constraint adaptation, surmount the static trade-off dilemma, endow a strong directional abscond mechanism to replace random perturbation, and actualize the inertia of directional exploration and the blind spots of solution exploitation. Twenty-three benchmark functions and six real-world engineering designs are employed to authenticate theoretical superiority and engineering practicability. The experimental results demonstrate that the MSBKA incorporates strong practicability and reliability to strengthen information interaction, restrain search stagnation, diminish convergence oscillation and fluctuation, facilitate globalized discovery and localized extraction, expedite convergence efficacy, ameliorate solution precision, and consolidate stability and robustness.

## 1. Introduction

Optimization scenarios are inherently concerned with selecting decision variables from a constrained feasible domain to achieve the extremum of a predetermined realistic function. The core principle employs system modeling, iterative search, and global convergence to abstract ambiguous requirements and intricate problems into quantifiable, solvable mathematical models, yielding scientific and objective optimal decision-making solutions and transcending the inherent subjectivity and boundedness of empirical judgment [1]. Traditional optimization methodologies are inherently circumscribed by the core algorithmic mechanisms and restrictive mathematical assumptions, which exhibit critical inadequacies of reliance on differentiability and higher-order information, attenuated engineering versatility or adaptability, global optimality deficiency, initialization sensitivity, inadequate robustness and generalizability, dimensionality disaster, restricted parallelization, narrow scalability and applicability, suboptimal local convergence, and formidable computational complexity. Swarm intelligence algorithms orchestrate emergent neighborhood interaction, reciprocal information exchange, and self-organizing collective evolution to explore high-dimensional solution spaces and asymptotically approach global optimality [2]. They encompass several distinctive characteristics such as broad generality, practical applicability, exemplary scalability, strong high-dimensional problem-solving ability, easy implementation and hybridizability enhancement, strong parallel search and stochastic perturbation, high compatibility and operational flexibility, and potent exploration and exploitation; these characteristics can be seen in Hannibal Barca optimization (HBO) [3], mother optimization algorithm (MOA) [4], Chinese pangolin optimization (CPO) [5], divine religions algorithm (DRA) [6], stellar oscillation optimization (SOO) [7], enzyme action optimization (EAO) [8], human evolutionary optimization algorithm (HEOA) [9], starfish optimization algorithm (SFOA) [10], tetragonula carbonaria builder bees (TGCOA) [11], H5N1 algorithm (H5N1) [12], mantis shrimp optimization algorithm (MShOA) [13], and the black-winged kite algorithm (BKA) [14].

The latest research on swarm intelligence mainly includes the following contents: (1) Collaborative multi-strategy mechanisms (e.g., historical collaborative, interval prediction, Cauchy mutation, quasi-opposition-based learning, Kent chaotic mapping, nearest neighborhood, population partitioning, dynamic subgroup), idiosyncratic encoding architectures (e.g., binary, real, integer, permutation, symbolic, complex, hybrid, quantum, gray, discrete, polar coordinate, fuzzy matrix, floating point encodings), and heterogeneous complementary approaches (e.g., falco peregrinus optimization, detective behavior algorithm, triangle centroid search algorithm, centered collision optimization, artificial lemming algorithm, dhole optimization algorithm) are utilized to harmonize exploration and exploitation, reinforce parallel convergence efficiency and holistic computational fidelity, and strengthen high-quality robustness and universality. (2) The research focus of swarm intelligence algorithms has shifted from verifying standard test functions to implementing complex engineering scenarios, and cross-domain adaptability has become the core indicator for evaluating algorithm performance. (3) The innovation direction of swarm intelligence is gradually focusing on emerging directions of distributed collaborative data-driven and multi-task optimization, a multi-agent system based on a large language model. The core requirement is to improve the stability and reproducibility of algorithms across massive datasets, large-scale clusters, and multi-task collaborative scenarios. Miao et al. consolidated the modified sand cat swarm optimization to recognize the dual-step infrastructure for forecasting the health situation of lithium-ion batteries, using decomposition and neuro-fuzzy networks; this method exhibited strong parallelism and consistency to realize seamless collaboration in exploration and exploitation, synchronously heighten convergence efficiency and solution accuracy, expedite convergence efficiency, and ameliorate solution precision [15]. Liu et al. articulated entropy-oriented multifaceted denoising and optimization-oriented neurological fuzzy modeling to resolve robust urban air quality index prediction for pollution management; this method exhibited strong practicality and superiority to maintain fortified simplicity and robustness, accelerate convergence speed and predictive efficiency, and promote calculation efficiency and solution quality [16]. Miao et al. extracted the modified fuzzy neural fluid dynamical system to resolve real-time path planning for unmanned aerial vehicles in complex three-dimensional dynamic environments; this method manipulated the reasonable configuration of globalized discovery and localized extraction, strengthened the flexibility and durability of complex systems, and attained the optimal real-time path [17].

Zhao et al. integrated opposition-based learning with a quasi-Newton strategy to demonstrate BKA’s complementary advantages and comprehensive collaboration; this method broadened population distribution heterogeneity, mitigated search stagnation, accelerated global exploration efficiency, and enhanced local exploitation accuracy [18]. Hui et al. constructed an intensified BKA to validate feasibility and practicability; this method demonstrated strong superiority and robustness in exploring solution space, materializing deep integration of synergistic complementarity and adaptability strategies, and eliminating the decline of population diversity [19]. Almutairi et al. explained the soft-rime pursuit modulation and parabolic extrapolation refinement to BKA for addressing renewable photovoltaic modeling and precise parameter identification; this method materialized excellent building simulation compatibility, strengthened population uniform distribution, and actualized faster search efficiency and higher estimation precision [20]. Li et al. extracted the ameliorative multi-strategy BKA to surmount the function optimization and engineering designs; this method manipulated the reasonable configuration of global detection and local mining, selected high-quality elite individuals, and strengthened search effectiveness and adaptability [21]. Guo et al. articulated the modified BKA to resolve the decomposition prediction cooperative model; this method enabled adaptive maintenance of population diversity, eschewed premature convergence and redundant computation, and achieved superior convergence efficiency and prediction precision [22]. Li et al. exploited the potentiated BKA based on Hammersley patterns, artificial lemming avoidance, Lévy flight, and trapezoidal variation to tackle non-lethal kinetic strike parameters; this method demonstrated powerful extensibility and competitiveness to exploit convergence precision, accelerate detection efficiency, and effectuate precise strikes on moving targets [23]. Zhu et al. employed the extended BKA to materialize the hazard estimation of cascading breakdowns; this method operated objective multi-dimensional evaluation metrics to accurately imitate the dynamic coupling capture of cascading faults, comprehensively realize collaborative optimization of risk assessment and preventive control, and strengthen the adaptability and robustness of complex systems [24]. Qi et al. formulated the reinforced BKA based on stagnation-associated diversification and adjustable weak guidance to recognize global optimization and fault detection; this method demonstrated emphatic practicality and noticeable superiority to monitor convergence disturbances, guide search direction, promote calculation efficiency and solution quality, and maintain fortified simplicity and robustness [25]. Du et al. advocated the complex-valued BKA for function optimization and engineering layouts; this method demonstrated superior parallelism and consistency, intensifying population distribution diversity, circumventing dimensional disasters, heightening information capacity and collaboration efficiency, and cultivating meticulous mining accuracy [26]. Nagarajan et al. executed the BKA to surmount the autism detection of privacy-preserved data; this method promoted detection accuracy and generalization ability, strengthened stringent privacy protection and data security, accelerated convergence speed and training efficiency, and heightened scalability and practicality [27]. Chen et al. reported the multi-strategy BKA to address unmanned aerial vehicle path planning; this method demonstrates powerful dynamic real-time response and replanning ability to promote the smoothness, safety, floatability and superiority of path quality and attain faster planning efficiency, stronger obstacle avoidance ability, superior optimization robustness, and higher convergence precision [28]. Lv et al. articulated the fractional-order BKA to seal it with unmanned aerial vehicle path planning for moving target search; this method heightens the prediction accuracy and anti-interference ability of dynamic target trajectories, effectively avoiding obstacle interference, maximizing target capture probability, and realizing finer and more adaptive search step size control [29]. Liao et al. consolidated the modified BKA to tackle global optimization; this method employed the collaborative exploit and information sharing of the multiple strategies to strengthen robustness and scalability, eschew premature convergence and search stagnation, and synchronously heighten convergence efficiency and solution accuracy [30]. Li et al. integrated the osprey optimization algorithm with horizontal and vertical directions to strengthen information exchange efficiency and search strategy diversity; this method realized seamless collaboration in depth and breadth, exploration and exploitation, and inheritance and innovation, demonstrating more stable and reliable convergence efficiency, solution accuracy, and robustness [31]. Although different variants of the BKA are used to solve function optimization and complex applications, this has verified its flexibility and practicability in global detection and local extraction, convergence efficiency, and solution precision. The BKA imitates the elevated-altitude circling exploration, fixed-point diving attack, and group cooperative migration of the black-winged kites to attain high-quality convergence precision. The BKA still needs to be further injected with general enhanced strategies to remedy deficiencies of the ponderous convergence speed, inadequate calculation accuracy, strong parameter sensitivity, serious dimension disaster, negative diversity maintenance, and restricted initial population quality. The no-free-lunch (NFL) method unequivocally clarifies the existence probability of a universal optimal algorithm for all optimization tasks, which will shift the focus of research and application from finding the best algorithm to designing the most appropriate algorithm, explicating population diversity, emphasizing problem relevance, and locating algorithmic value.

The distinctive contributions of the MSBKA are highlighted as follows: (1) The ranking-based differential mutation, simplex method and elite opposition-based learning strategy are integrated into the BKA to address the function of global optimization and constrained engineering applications. (2) The ranking-based differential mutation stabilizes convergence efficiency, eliminates homogenization competition, promotes population guidance, sustains population diversity, and strengthens information interaction. (3) The simplex method consolidates local excavation, ameliorates calculation precision, diminishes parameter sensitivity, accelerates accurate convergence, and eschews search stagnation. (4) The elite opposition-based learning strategy expands the detection area, maintains population diversity, restrains premature convergence, fortifies solution quality, and promotes intelligent detection. (5) The MSBKA is contrasted with other newly publicized, extensively referenced, and extremely competitive algorithms, such as HBO, MOA, CPO, DRA, SOO, EAO, HEOA, SFOA, TGCOA, H5N1, MShOA and BKA. The MSBKA is validated against the twenty-three benchmark functions and six engineering designs. (6) The MSBKA not only demonstrates strong superiority and practicality to achieve synergistic complementarity of strategic advantages and harmonize globalized discovery and localized extraction, but also demonstrates strong adaptability and feasibility to expedite convergence efficacy, ameliorate solution precision, and consolidate stability and robustness.

The subsequent sections of this article are outlined as follows. Section 2 articulates the BKA. Section 3 furnishes the MSBKA. Section 4 elucidates simulation results and experimental analysis for benchmark functions. Section 5 furnishes the MSBKA for accomplishing the constrained engineering applications. Section 6 recapitulates the conclusion and future research.

## 2. BKA

The BKA reconciles Cauchy mutation and Leader selection to preserve exploratory diversity, accelerate convergence efficiency, mitigate search stagnation, approach the optimal vicinity, maintain population directionality, and reinforce fault tolerance and stability margins. The BKA simulates high-altitude circling exploration, fixed-point diving attack and group cooperative migration of the black-winged kites to facilitate optimal population distribution and cooperative evolution, harmonize exploratory divergence and exploitative convergence, and ascertain the globally optimal solution.

### 2.1. Initialization Population

Randomly initializing the population is performed to achieve a uniform distribution of candidate solutions and to sustain population diversity. The initialization matrix BK is decomposed as

(1) $$BK=\left[\begin{array}{cccc} BK_{1,1} & BK_{1,2} & \ldots & BK_{1,dim}\\ BK_{2,1} & BK_{2,2} & \ldots & BK_{2,dim}\\ \vdots & \vdots & \vdots & \vdots \\ BK_{pop,1} & BK_{pop,2} & \ldots & BK_{pop,dim} \end{array}\right]$$

where pop constitutes the population multiplicity, dim constitutes the problem dimensionality, and $BK_{i,j}$ constitutes *jth* dimension of *ith* solution. The $X_{i}$ is decomposed as

(2) $$X_{i}=BK_{lb}+rand(BK_{ub}-BK_{lb})$$

where i∈[1,pop], $BK_{lb}$ and $BK_{ub}$ constitute the boundary constraints, $rand\in \left[0,1\right]$.

The optimal pathfinder $X_{L}$ is decomposed as

(3) $$f_{best}=\min(f(X_{i}))$$

(4) $$X_{L}=X(find(f_{best}==f(X_{i})))$$

### 2.2. Meticulous Attacking Behavior (Fine-Grained Exploitation Phase)

**Figure 1:**
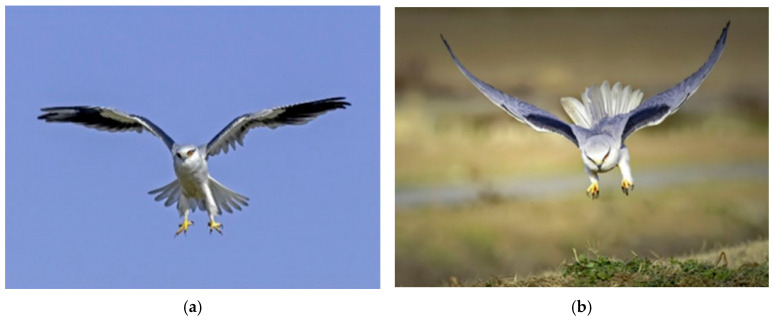
(**a**) Sustained hovering. (**b**) High-velocity dive attack on prey.

**Figure 2:**
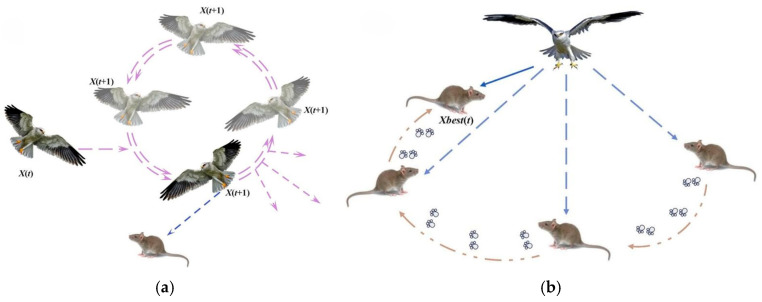
(**a**) Aerial hovering, awaiting attack prey. (**b**) Aerial hovering, facilitate prey detection.

The BKA orchestrates posture adjustment, precise diving, and hovering approximation to achieve efficient population traversal and high-quality region locking synergistically. The BKA is distinguished by explicit biomimetic guidance and dynamic adaptability, exhibiting remarkable superiority in efficiently, accurately, and stably attaining global optima. Figure 1a characterizes the sustained hovering of the black-winged kite. Figure 1b characterizes the high-velocity dive attack on prey. Figure 2a characterizes the black-winged kite sustaining aerial hovering, awaiting attack prey. Figure 2b characterizes the black-winged kite’s ability to sustain aerial hovering, facilitating prey detection.

The meticulous attacking is decomposed as

(5) $$y_{t+1}^{i,j}=\left\{\begin{array}{cc} y_{t}^{i,j}+n(1+\sin(r))\times y_{t}^{i,j} & p<r\\ y_{t}^{i,j}+n\times (2r-1)\times y_{t}^{i,j} & p\geq r \end{array}\right.$$

(6) $$n=0.05\times e^{-2\times {\left(\frac{t}{T}\right)}^{2}}$$

where $y_{t}^{i,j}$ and $y_{t+1}^{i,j}$ constitute the positions of the *ith* black-winged kite in the *jth* dimension, $r\in \left[0,1\right]$ is the probability threshold p=0.9, and T constitutes the maximum iteration.

When p<r, the hovering attack serves as a biomimetic analog to the black-winged kite’s long-range hovering observation and gradual prey approach behavior, which harnesses its expansive exploration range and pronounced stochasticity to thoroughly scan potential high-quality regions, thereby circumventing the local optima predicament induced by initial population clustering.

When p≥r, the diving attack epitomizes the black-winged kite’s strategy of executing a close-range dive to achieve pinpoint prey capture, leveraging a constrained search range and heightened precision to meticulously exploit promising regions, progressively converging on the global optimum.

### 2.3. Swarm Migration Behavior (Coarse-Grained Exploration Phase)

**Figure 3:**
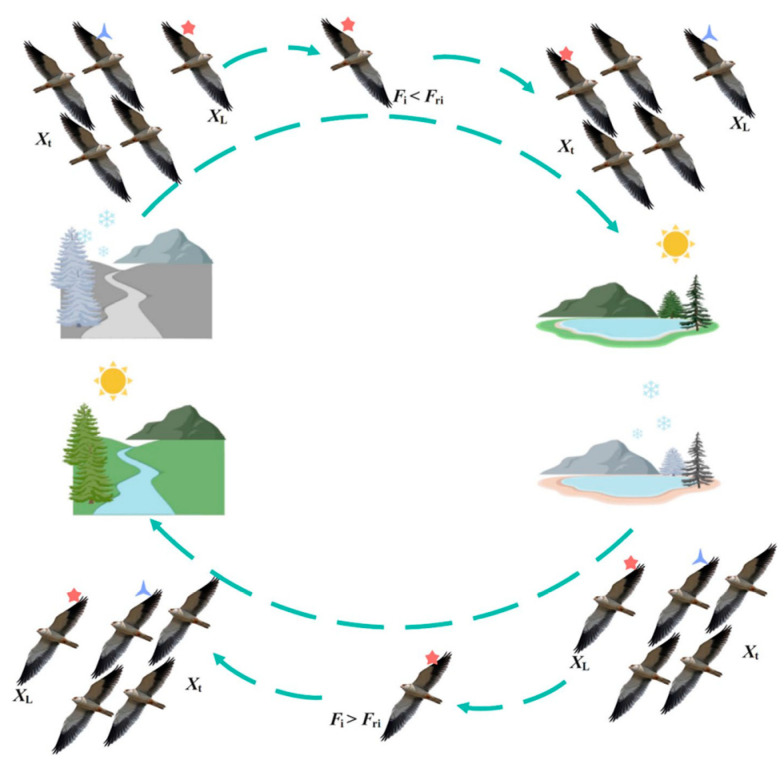
The migration strategy transitions of the black-winged kites.

The swarm migration behavior of black-winged kites mirrors their natural response to food scarcity and unfavorable environmental conditions, prompting them to undertake long-distance flights to new habitats in search of resources. In the algorithmic framework, this behavior is abstracted as large-scale population repositioning and strategic reconfiguration of individual populations, which essentially establishes durability and sustainability. Figure 3 characterizes the migration strategy transitions of the black-winged kites.

The swarm migration is decomposed as

(7) $$y_{t+1}^{i,j}=\left\{\begin{array}{ll} y_{t}^{i,j}+C(0,1)\times (y_{t}^{i,j}-L_{t}^{j}) & F_{i}<F_{ri}\\ y_{t}^{i,j}+C(0,1)\times (L_{t}^{j}-m\times y_{t}^{i,j}) & F_{i}\geq F_{ri} \end{array}\right.$$

(8) m=2×sin(r+π/2)

where $L_{t}^{j}$ constitutes the elite leader, $F_{i}$ constitutes the current position, $F_{ri}$ constitutes the refreshed position, and C(0,1) constitutes the Cauchy mutation.

If the fitness of the current population exceeds that of the stochastic population, the leader perpetuates the directional guidance toward the destination and the superior individuals consistently orchestrate the search trajectory and systematically guide the population toward convergence in high-potential areas.

If the fitness of the current population is inferior to that of the stochastic population, the leader abdicates the directive role and merges with the migrating population; the insufficient leadership does not compromise global search capability.

The BKA orchestrates the heavy-tailed distribution of Cauchy mutations with a dynamic leader-switching mechanism to facilitate collaborative population migration, boost population diversity, and strengthen detection efficiency and convergence accuracy. The probability density of the univariate Cauchy distribution is decomposed as

(9) $$f(x,\delta ,\mu )=\frac{1}{\pi }\frac{\delta }{\delta^{2}+{\left(x-\mu \right)}^{2}}\;-\infty <x<\infty$$

where δ=1, μ=0.

The normalized probability density function is decomposed as

(10) $$f(x,\delta ,\mu )=\frac{1}{\pi }\frac{\delta }{x^{2}+1}\;-\infty <x<\infty$$

Algorithm 1 encapsulates the pseudocode of BKA.
**Algorithm 1** BKA**Begin****Step 1.** Initialize BKA population $X_{i}\;(i=1,2,\ldots ,n)$
**Step 2.** Corroborate fitness of each black-winged kite    Retrieve $X_{best}$$\;\mathrm{and}\;F_{best}$
**Step 3. while** (t<T) do     **for** each black-winged kite **do**     /*Meticulous attacking phase*/       **if**
p<r
        Recalibrate the attacking location via Equation (5)         
$y_{t+1}^{i,j}=y_{t}^{i,j}+n\left(1+sin\left(r\right)\right)\times y_{t}^{i,j}$
       **else if do**
        Recalibrate attacking location via Equation (5)         $y_{t+1}^{i,j}=y_{t}^{i,j}+n\times \left(2r-1\right)\times y_{t}^{i,j}$
       **end if**
     /*Swarm migration phase*/       **if** Fi<Fri        Recalibrate migration location via Equation (7)         $y_{t+1}^{i,j}=y_{t}^{i,j}+C\left(0,1\right)\times \left(y_{t}^{i,j}-L_{t}^{j}\right)$
       **else if do**
        Recalibrate migration location via Equation (7)         $y_{t+1}^{i,j}=y_{t}^{i,j}+C\left(0,1\right)\times \left(L_{t}^{j}-m\times y_{t}^{i,j}\right)$
       **end if**     /* Handpick the fittest individual*/       **if** $y_{t+1}^{i,j}<L_{t}^{j}$         $X_{best}=y_{t+1}^{i,j},\;F_{best}=f\left(y_{t+1}^{i,j}\right)$
       **else if do**
         $X_{best}=L_{t}^{j},\;F_{best}=f\left(L_{t}^{j}\right)$
       **end if**
     Enforce boundary constraints by detecting and correcting violations     Corroborate fitness of each black-winged kite     $\mathrm{Recalibrate}\;X_{best}$ if there is a superior black-winged kite     **end for**
     t=t+1
    **end while**
    Retrieve $X_{best}$$\;\mathrm{and}\;F_{best}$
**End**

## 3. MSBKA

To compensate the deficiencies of the ponderous convergence speed, inadequate calculation accuracy, strong parameter sensitivity, serious dimension disaster, negative diversity maintenance, and restricted initial population quality, the MSBKA with the ranking-based differential mutation, simplex method, and elite opposition-based learning strategy not only actualizes synergetic complementary and rationalized configuration to reduce the oscillation and fluctuation of the optimal solution, but also integrates deep exploitation and breadth exploration to expedite convergence efficacy, facilitate solution precision, and consolidate stability and robustness.

### 3.1. Ranking-Based Differential Mutation

The ranking-based differential mutation employs sorting, allocation, and selection probability mechanisms to achieve precise quantitative allocation between exploration and exploitation, thereby strengthening search directionality and purposefulness, maintaining dynamic population heterogeneity, establishing fully adaptive pressure regulation, and attaining structural modularity and paradigm universality [32]. The sorting allocation orchestrates precise population categorization, clarifies individual update positioning, eliminates scale differences, furnishes transparent population status information, and translates absolute fitness into relative ranking levels. The architectural principles of sorting allocation are highlighted as follows: (1) Priority selection prioritizes individual fitness, ensuring that high-quality individuals are assigned superior rankings, thereby maintaining exploitation precision. (2) The diversity principle takes into account individual distribution disparities, maintains population heterogeneity, and suppresses premature convergence. (3) The operability principle features concise hierarchical logic, effortless parameter tuning, low computational overhead, and broad algorithmic compatibility. The sorting allocation $R_{i}$ is decomposed as

(11) $$R_{i}=N_{p}-i,\;i=1,2,\ldots ,N_{p}$$

where $N_{p}$ constitutes the population multiplicity. 

The selection probability allocates rational resource distribution, prioritizes individual update priorities, enforces survival of the fittest, focuses computational effort on high-potential regions, and directs the search trajectory toward optimality. The architectural principles of selection probability are highlighted as follows: (1) The level adaptation principle establishes a monotonically positive correlation between selection probability and individual hierarchical status, ensuring that high-quality individuals are prioritized in accessing update resource. (2) The diversity principle maintains robust perturbations, retains the selection probabilities for individuals of all quality strata (excellent, ordinary, and inferior), averts outright elimination, perpetuates population heterogeneity, and mitigates premature convergence. (3) The normalization principle confers rationality and operability upon probability allocation, thereby facilitating the execution of subsequent update strategies. The selection probability $P_{i}$ is constructed as

(12) $$p_{i}=\frac{R_{i}}{N_{p}},\;i=1,2,\ldots ,N_{p}$$

The differential mutation employs sorting probability to select high-quality individuals as base vectors preferentially, steering the search toward promising areas, stabilizing convergence efficiency, promoting population guidance, reducing disorderly fluctuations, and sustaining stability and reliability. Algorithm 2 encapsulates the ranking-based differential mutation of “DE/rand/1”.
**Algorithm 2** Ranking-based differential mutation of “DE/rand/1”**Begin**Sort the population and distribute the selection probability $P_{i}$
Randomly select $r_{1}\in \left\{1,N_{p}\right\}$ {base vector index}**while** $rand>p_{r_{1}}\;or\;r_{1}==i$Randomly select $r_{1}\in \left\{1,N_{p}\right\}$
**end**Randomly select $r_{2}\in \left\{1,N_{p}\right\}$ {terminal vector index}**while** $rand>p_{r_{2}}\;or\;r_{2}==r_{1}\;or\;r_{2}==i$Randomly select $r_{2}\in \left\{1,N_{p}\right\}$
**end**Randomly select $r_{3}\in \left\{1,N_{p}\right\}$ {starting vector index}**while** $r_{3}==r_{2}\;or\;r_{3}==r_{1}\;or\;r_{3}==i$Randomly select $r_{3}\in \left\{1,N_{p}\right\}$
endEnd

### 3.2. Simplex Method

**Figure 4:**
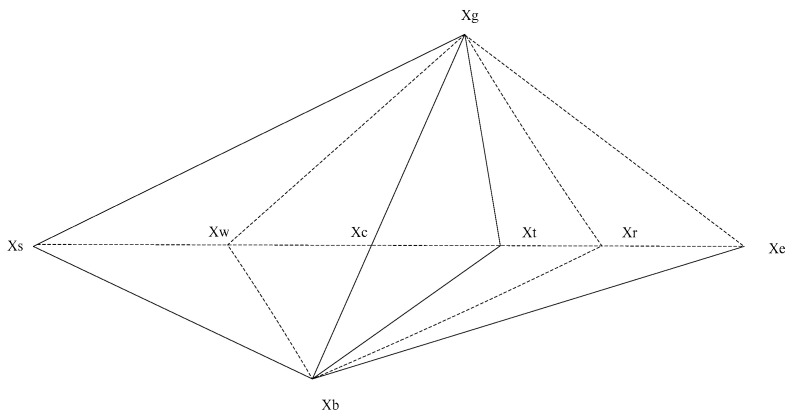
Simplex method schematic.

The simplex method is distinguished by gradient independence, computational parsimony, and a structured search paradigm, which evaluates solution quality solely based on the ordinal ranking of fitness values and operates independently of the differentiability or continuity of the objective function [33]. The simplex method constructs a geometric convex polyhedron from selected high-quality individuals, iteratively refines the vertices through the geometric operations of reflection, expansion, compression and contraction, and progressively converges toward the global optimal solution. Figure 4 characterizes the simplex polyhedron transformation schematic.

**Step 1.** Quantify and sort fitness according to each geometric vertex; corroborate the fitness $f(X_{g})$, $f(X_{b})$ and $f(X_{s})$ of the highest-quality vertice $X_{g}$, intermediate-quality vertice $X_{b}$, and lowest-quality vertice $X_{s}$ separately, which locates the pessimal solution, ensures optimization quality, and promotes iteration efficiency. 

**Step 2.** Centroid calculation: Corroborate the geometric centroid $X_{c}$; $X_{c}$ is the central direction of the current high-quality search region to guide extended geometric maneuvers, maintain search directionality and eschew stochastic wandering.

(13) $$X_{c}=\frac{X_{g}+X_{b}}{2}$$

**Step 3.** Reflection operation: Generate a refraction vertex $X_{r}$ by extending the line from the intermediate-quality vertice $X_{b}$ to the geometric centroid $X_{c}$ and corroborate fitness $f(X_{r})$ and reflection coefficient α=1.

(14) $$X_{r}=X_{c}+\alpha (X_{c}-X_{s})$$

If $f(X_{r})<f(X_{g})$, the refraction vertex $X_{r}$ is superior to the highest-quality vertice $X_{g}$; the refraction direction evidences a superior solution, necessitates the expansion operation, magnifies the directional advantage, and accelerates convergence efficiency. The next step is to perform the expansion operation.

If $f(X_{g})\leq f(X_{r})\leq f(X_{s})$ and the refraction vertex $X_{r}$ is superior to the lowest-quality vertice $X_{s}$ and inferior to the highest-quality vertice $X_{g}$, then the refraction direction is effective and trustworthy. Replace the lowest-quality vertice $X_{s}$ with the refraction vertex $X_{r}$, then execute the next iteration. 

If $f(X_{r})>f(X_{s})$, the refraction vertex $X_{r}$ is inferior to the lowest-quality vertice $X_{s}$, the refraction direction is invalid. The next step is to perform the compression operation.

**Step 4.** Expansion operation: When the refraction vertex $X_{r}$ is superior to the highest-quality vertice $X_{g}$, which further extends along the reflection direction, accelerates convergence speed, and approaches the optimal solution. Generate an expansion vertex $X_{e}$ by extending the line from the geometric centroid $X_{c}$ to the refraction vertex $X_{r}$, corroborate fitness $f(X_{e})$, and the expansion coefficient γ=2.

(15) $$X_{e}=X_{c}+\gamma (X_{r}-X_{c})$$

If $f(X_{e})<f(X_{g})$, replace the lowest-quality vertice $X_{s}$ with an expansion vertex $X_{e}$. Otherwise, replace the lowest-quality vertice $X_{s}$ with the refraction vertex $X_{r}$.

**Step 5.** Compression operation: When the refraction vertex $X_{r}$ is inferior to the lowest-quality vertice $X_{s}$, it further shrinks towards the geometric centroid and conducts a refined search. Generate a compression vertex $X_{t}$ by extending the line from the lowest-quality vertice $X_{s}$ to the geometric centroid $X_{c}$, corroborate fitness $f(X_{t})$, and compression coefficient β=0.5.

(16) $$X_{t}=X_{c}+\beta (X_{s}-X_{c})$$

If the $f(X_{t})<f(X_{s})$, replace the lowest-quality vertice $X_{s}$ with compression vertex $X_{t}$. Otherwise, replace the lowest-quality vertice $X_{s}$ with the refraction vertex $X_{r}$.

**Step 6.** Contraction operation: When the compression operation is ineffective and the compression vertex $X_{t}$ is inferior to the lowest-quality vertice $X_{s}$, the operation has a large search range and poor overall quality to abandon ineffective searches; focus on high-quality areas and avoid resource waste. Generate a contraction vertex $X_{w}$ by shrinking all vertices towards the highest-quality vertice $X_{g}$, corroborate fitness $f(X_{w})$, and compression coefficient δ=0.5.

(17) $$X_{w}=X_{c}-\delta (X_{s}-X_{c})$$

Algorithm 3 encapsulates the pseudocode of the simplex method.
**Algorithm 3** Simplex method**Input:** The highest-quality vertice $X_{g}$, intermediate-quality vertice $X_{b}$, lowest-quality vertice $X_{s}$
**Output:** new candidate solution $X_{new}$$X_{c}=\frac{X_{g}+X_{b}}{2}$   (Centroid calculation)$X_{r}=X_{c}+\alpha \times (X_{c}-X_{s})$   (Reflection operation)**if** $f(X_{r})<f(X_{g})$ **then**   (Expansion operation)   $X_{e}=X_{c}+\gamma (X_{r}-X_{c})$
   **If** $f(X_{e})<f(X_{g})$ **then**     $X_{s}=X_{e}$
   **else**
     $X_{s}=X_{r}$
   **end if**
**end if****if** $f(X_{s})<f(X_{r})$ **then**   (Compression operation)   $X_{t}=X_{c}+\beta (X_{s}-X_{c})$
   **if** $f(X_{t})<f(X_{s})$ **then**      $X_{s}=X_{t}$
   **end if**
**end if****if** $f(X_{g})\leq f(X_{r})\leq f(X_{s})$ **then**   (Contraction operation)   $X_{w}=X_{c}-\delta (X_{s}-X_{c})$
    **if** $f(X_{w})<f(X_{s})$ **then**      
$X_{s}=X_{w}$
   **else**
      $X_{s}=X_{r}$
   **end if**
**end if**$X_{new}=X_{s}$

### 3.3. Elite Opposition-Based Learning Strategy

The elite opposition-based learning strategy confines the opposition solutions to elite individuals within the current population, selects the best individuals from the union of original and opposition elites, and achieves high-quality population renewal with low computational expenditure [34]. This strategy can eliminate population homogeneity, restrain premature convergence, broaden discovery scope, fortify the quality of initial and iterative solutions, guide intelligent search direction, and enrich population variety. The current elite solution is $X_{Elite}=(X_{Elite,1},X_{Elite,2},\ldots ,X_{Elite,D})$; the search agent is $X_{i}=(X_{i,1},X_{i,2},\ldots ,X_{i,D})$; and the reverse search agent is $X_{i}^{\prime }=(X_{i,1}^{\prime },X_{i,2}^{\prime },\ldots ,X_{i,D}^{\prime })$.

(18) $$X_{i,j}^{\prime }=k\cdot (lb_{j}+ub_{j})-X_{e,j},\;i=1,2,\ldots ,N;\;j=1,2,\ldots ,D$$

where N constitutes the population multiplicity, D constitutes the problem dimensionality, k∈(0,1), and lb and ub constitute the dynamic boundary constraints.

(19) $$lb_{j}=\min(X_{i,j}),\;ub_{j}=\max(X_{i,j})$$

The reverse search agent exceeds the prescribed dynamic boundaries; $X_{i,j}^{\prime }$ is decomposed as

(20) $$X_{i,j}^{\prime }=rand(lb_{j},ub_{j}),\;if\;X_{i,j}^{\prime }<lb_{j}\;or\;X_{i,j}^{\prime }>ub_{j}$$

### 3.4. The Solution Process of MSBKA

**Figure 5:**
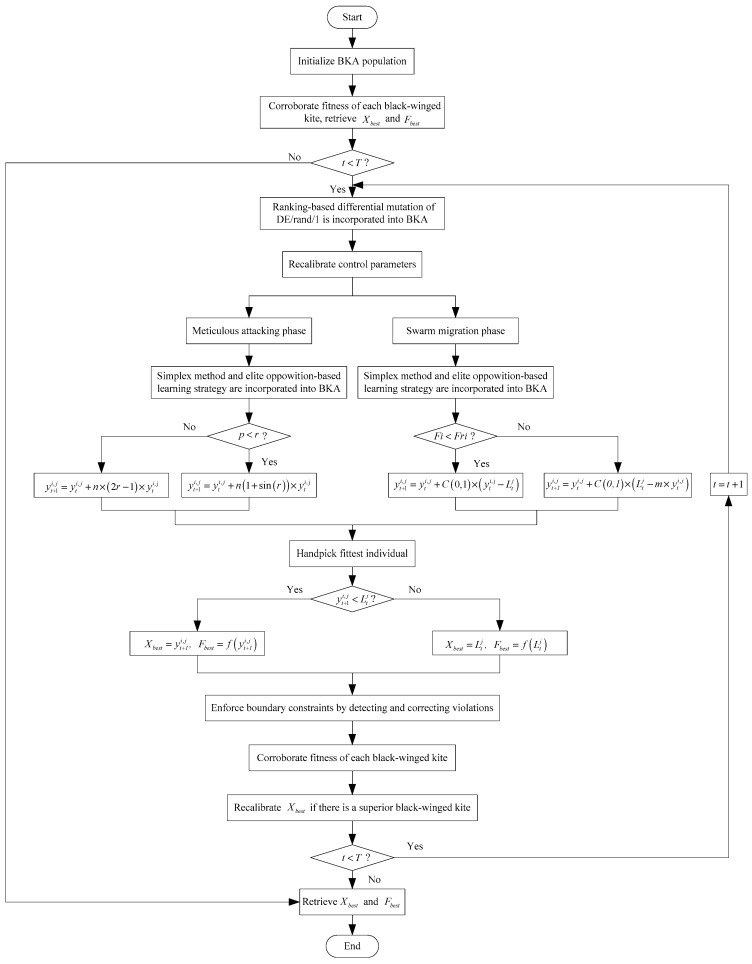
Flowchart of MSBKA.

Algorithm 4 encapsulates the pseudocode of MSBKA. Figure 5 characterizes the flowchart of the MSBKA.
**Algorithm 4** MSBKA**Begin****Step 1.** Initialize BKA population $X_{i}\;(i=1,2,\ldots ,n)$
**Step 2.** Corroborate fitness of each black-winged kite    Retrieve $X_{best}$ and $F_{best}$
**Step 3. while** (t<T) do    Ranking-based differential mutation of “DE/rand/1” is incorporated into BKA    **for** each black-winged kite **do**    /*Meticulous attacking phase*/    Simplex method and elite opposition-based learning strategy are incorporated into BKA      **if** p<r         Recalibrate the attacking location via Equation (5)         $y_{t+1}^{i,j}=y_{t}^{i,j}+n\left(1+sin\left(r\right)\right)\times y_{t}^{i,j}$
      **else if do**
         Recalibrate attacking location via Equation (5)         $y_{t+1}^{i,j}=y_{t}^{i,j}+n\times \left(2r-1\right)\times y_{t}^{i,j}$
      **end if**
    /*Swarm migration phase*/      Simplex method and elite opposition-based learning strategy are incorporated into BKA      **if** Fi<Fri         Recalibrate migration location via Equation (7)         $y_{t+1}^{i,j}=y_{t}^{i,j}+C\left(0,1\right)\times \left(y_{t}^{i,j}-L_{t}^{j}\right)$
      **else if do**
         Recalibrate migration location via Equation (7)         $y_{t+1}^{i,j}=y_{t}^{i,j}+C\left(0,1\right)\times \left(L_{t}^{j}-m\times y_{t}^{i,j}\right)$
      **end if**
    /* Handpick the fittest individual*/      **if** $y_{t+1}^{i,j}<L_{t}^{j}$         $X_{best}=y_{t+1}^{i,j},\;F_{best}=f\left(y_{t+1}^{i,j}\right)$
      **else if do**
         $X_{best}=L_{t}^{j},\;F_{best}=f\left(L_{t}^{j}\right)$
      **end if**
      Enforce boundary constraints by detecting and correcting violations      Corroborate fitness of each black-winged kite       Recalibrate $X_{best}$ if there is a superior black-winged kite      **end for**
      t=t+1
    **end while**
    Retrieve $X_{best}$ and $F_{best}$
**End**

## 4. Simulation Results and Experimental Analysis for Benchmark Functions

### 4.1. Experimental Setup

All demonstration platforms were accomplished on a machine incorporating a 64-bit Windows 11 operating system, a 12th Gen Intel Core i9-12900HX processor (2.30 GHz), 16 GB of RAM, a 4 TB storage device, and a dedicated 16 GB GPU. All comparative methods were coded and executed in MATLAB R2022b.

### 4.2. Benchmark Functions

Benchmark functions are methodically categorized into three categories: unimodal functions $f_{1}-f_{7}$, multimodal functions $f_{8}-f_{13}$, and fixed-dimensional multimodal functions $f_{14}-f_{23}$. The unimodal functions are employed to scrutinize the convergence dynamics and solution consistency and converge to the unique global optimum. The multimodal functions are designed to circumvent local optima effectively, accurately identify the global optimum or approximate near-optimal solutions, and exhibit a synergistic balance between exploratory breadth and exploitative depth. The fixed-dimensional multimodal functions are utilized to estimate robustness, accuracy, and reproducibility in the fixed complexity solution space, accurately locate multiple optimal solutions, efficiently maintain the convergence stability and detection accuracy, and verify the adaptability and scalability. Table 1 emphasizes the benchmark functions.

### 4.3. Parameter Configuration

To authenticate the design rationality and experimental strictness, the MSBKA is contrasted with HBO, MOA, CPO, DRA, SOO, EAO, HEOA, SFOA, TGCOA, H5N1, MShOA and BKA. The parameter configuration and sensitivity analysis are summarized as follows: (1) These parameter selections are based on empirical fixation and universal inheritance principles, which have been rigorously screened and inherited from representative empirical values in the original literature. The small fluctuations of the parameter values have little impact on the convergence speed, computational accuracy, and stability of the MSBKA. The empirical fixed values have been extensively simulated to verify the optimal robustness. They have strong robustness, reliability, inheritance, and practicality to follow the principles of hierarchy and functionality. The necessity of repetitive experimental verification is relatively low. Modifying these parameters would undermine the mathematical foundation, convergence consistency, standardization and rationality of the MSBKA. (2) The MSBKA adopts the synergistic complementarity of ranking-based differential mutation, the simplex method, and the elite opposition-based learning strategy to essentially diminish parameter sensitivity and provide core support. The ranking-based differential mutation utilizes the fitness and spatial distribution of individual populations for comprehensive sorting, dynamically guiding the direction of population iteration, which effectively offsets the convergence speed’s fluctuations caused by small parameter fluctuations, avoids invalid iterations by parameter value deviations, and ensures the orderliness of population renewal. The MSBKA utilizes the reflection, expansion, and contraction of the simplex method to strengthen local mining efficiency, ameliorate solution accuracy, abate parameter sensitivity, eschew local optimal traps, and accelerate accurate convergence. The elite opposition-based learning strategy performs reverse mapping on contemporary elite individuals, generating reverse elite individuals to supplement population diversity, elevates the quality of initial and iterative solutions, and guides intelligent search direction. (3) These parameters have inherent fault tolerance, a dynamic compensation mechanism, and collaborative architecture to diminish single-point vulnerability, equalize exploration and exploitation, ensure comprehensive superiority, and reuse recognized experience values in terms of convergence speed, solution quality, stability and robustness. The ranking-based differential mutation, simplex method, and elite opposition-based learning strategy have strong optimization potential for complete decoupling, complementary intersection, and functional orthogonality. There is no direct coupling path or interaction channel between parameters. Even if individual parameters fluctuate within a reasonable range, it will not lead to the failure of other strategies.

HBO: ratio α=2/3, normal distribution N(0,1), coefficient Coef=0.94.

MOA: stochastic elements rand∈[0,1], $rand(2)\in \left\{1,2\right\}$.

CPO: stochastic elements $r_{1}\in [0,1]$, $r_{2}\in [0,1]$, rand∈[0,1], u∈[0,1], v∈[0,1], aroma concentration $C_{M}\in [0.2,0.7]$, source of aroma Q=100, aroma diffusion coefficient Dc=0.6, levy factor β=1.5, constant element s=0.01.

DRA: belief profile selection probability BPSP=0.5, miracle probability MP=0.5, proselytism probability PP=0.9, reward or penalty probability RP=0.2.

SOO: stochastic elements $r_{1}\in [0,1]$, $r_{2}\in [0,1]$, $r_{3}\in [0,1]$, scaling factor S∈[0,2], refresh probability $r_{j}=0.5$.

EAO: stochastic elements r∈[0,1], ρ∈[0,1], $sc_{1}\in [0.1,1]$, $sc_{2}\in [0.1,1]$, enzyme concentration EC=0.1.

HEOA: stochastic elements rand∈[0,1], r∈[0,1], R∈[0,1], δ∈[100,2000], constant element γ=1.5, evaluation element A=0.6.

SFOA: stochastic elements r∈(0,1), $r_{1}\in (0,1)$, $r_{2}\in (0,1)$, θ∈(0,π/2), $a_{1}\in (-\pi ,\pi )$, $A_{1}\in (-1,1)$, $A_{2}\in (-1,1)$.

TGCOA: thermal conductivity k=0.03, wing flapping amplitude A∈[0.2,0.3], Wings flapping frequency α=50, stochastic elements β∈[0,1], λ∈[0,1], f∈(0,1).

H5N1: infection probability $P_{1}=0.8$, mutation probability $P_{2}=0.85$.

MShOA: stochastic elements rand∈[0,1], $PTI\in \left\{1,2,3\right\}$, D∈[−1,1], angular strike motion θ∈[π,π/2], scaling factor k∈[0,0.3].

BKA: stochastic elements rand∈[0,1], r∈[0,1], constant elements p=0.9, δ=1, μ=0, Cauchy mutation C∈(0,1).

MSBKA: stochastic elements rand∈[0,1], r∈[0,1], constant elements p=0.9, δ=1, μ=0, Cauchy mutation C∈(0,1), scaling factor F=0.7, reflection coefficient α=1, expansion coefficient β=1.5, compression coefficient γ=0.2, contraction coefficient δ=0.5, stochastic element k∈(0,1), elite probability χ=0.2.

### 4.4. Simulation Results and Experimental Analysis

To comprehensively and scientifically implement the impartiality and trustworthiness of the verification results of each algorithm, the population size is N=50, the maximum iteration is T=1000, and independent reproduction is R=30. The optimal value (Best), worst value (Worst), mean value (Mean), standard deviation (Std), convergence analysis, boxplot analysis, and Wilcoxon rank-sum test are comprehensive absolute core statistical indicators, which can execute a multi-level and multi-dimensional mechanism to disinterestedly measure detection precision, performance robustness, convergence stability, comprehensive optimization, superiority and practicality.

Table 2 emphasizes the numerical statistics results of MSBKA and mainstream algorithms on benchmark functions. Multiple mainstream algorithms manipulate horizontal comparison and the fittest paradigm to recognize global optimization; the attempt is to imitate the collaborative search and information exchange of biological populations to equalize exploratory breadth and exploitative depth, recognize the rationality and superiority, materialize universality and scalability, and actualize stationary global or approximate optimal solutions for complex functions. For unimodal functions $f_{1}-f_{7}$, the MSBKA, DRA, EAO, SFOA, TGCOA, H5N1 and MShOA have all demonstrated theoretically optimal convergence consistency, consummating statistical robustness for $f_{1}$, $f_{2}$, $f_{3}$ and $f_{4}$. The optimal values, worst values, mean values, and standard deviations of the MSBKA significantly outperform those of the HBO, MOA, CPO, SOO, HEOA and BKA; the MSBKA not only accurately materializes the globally accurate extremum solution and demonstrates extremely high solution quality, but also exhibits comprehensive consistency in multiple independent repeated experiments. This fully confirms the distinguished responsibility and completeness and materializes the ultimate performance. For $f_{5}$, the optimal value, worst value and mean value of the MSBKA are second only to HBO, MOA, CPO, DRA, HEOA, and H5N1 but ahead of SOO, EAO, SFOA, TGCOA, MShOA and BKA. The MSBKA demonstrates extraordinary fault tolerance and universality to maintain population diversity, construct a high-quality solution space distribution, and achieve multi-dimensional superiority. The standard deviation of the MSBKA is superior to that of HBO and CPO but inferior to MOA, DRA, SOO, EAO, HEOA, SFOA, TGCOA, H5N1, MShOA and BKA. The MSBKA exhibits insufficient stability to trigger significant fluctuations, high dispersion degree, search imbalance and uncontrollability. For $f_{6}$ and $f_{7}$, the quantitative assessment metrics of the MSBKA are stronger than those of the HBO, CPO, DRA, EAO, HEOA, SFOA, TGCOA, MShOA and BKA and weaker than those of the MOA, SOO, and H5N1. The MSBKA exhibits excellent convergence quality in both the optimal boundary and the overall distribution, materializes ultimate convergence accuracy and extraordinary stability performance, equalizes the synergistic effects of multiple strategies mechanisms, locks confidence intervals, and eliminates search fluctuations. For multimodal functions $f_{8}-f_{13}$, the quantitative assessment metrics of the MSBKA, MOA, CPO, DRA, SOO, EAO, SFOA, TGCOA, H5N1, MShOA and BKA maintain strictly high consistency of numerical values with zero fluctuation; they comprehensively surpass HBO and HEOA for $f_{9}$ and $f_{11}$. The MSBKA actualizes collaborative complementarity of multiple strategies to comprehensively strengthen detection efficiency and mining precision, highlight global exploration stability, attain precise positioning of exact solution, diminish convergence oscillation, and demonstrate strong robustness and reproducibility. For $f_{8}$, $f_{12}$ and $f_{13}$, the comprehensive optimal values, worst values, mean values, and standard deviations of the MSBKA are superior to those of the HBO, CPO, and H5N1 but inferior to those of the MOA, DRA, SOO, EAO, HEOA, SFOA, TGCOA, MShOA and BKA. The MSBKA manipulates the population sorting mutation operator, local meticulous excavation, which is an efficacious escape mechanism to achieve centralized and extensive search, strengthens detection efficiency, downgrades oscillation disturbance, consolidates consistency and reliability, arrests population iteration stagnation, and reconstructs high-quality solutions. For $f_{10}$, the optimal values, worst values, and mean values of the MSBKA, MOA, CPO, DRA, EAO, HEOA, SFOA, TGCOA, H5N1, MShOA and BKA exhibit preferable superiority and reliability to maintain numerical consistency. The MSBKA demonstrates strong superiority and repeatability to refine population structure, accurately screen elites, eliminate inferior individuals, improve optimization efficiency, avoid local optimal traps, reduce ineffective searches, and decrease redundant calculations. The standard deviation of the MSBKA successfully surpasses that of the HBO and SOO, and the MSBKA demonstrates excellent stability and robustness to eliminate initial fluctuations, reduce dispersion, maintain population diversity, and achieve smooth convergence. For fixed-dimensional multimodal functions $f_{14}-f_{23}$, the MSBKA, HBO, MOA, SFOA, H5N1 and BKA maintain the equivalent measurement level and exhibit a theoretically optimal deterministic convergence solution for $f_{14}$, $f_{15}$ and $f_{17}$. The quantitative assessment metrics of the MSBKA achieved comprehensive superiority over those of the CPO, DRA, SOO, EAO, HEOA, TGCOA and MshOA. The MSBKA exploits the difference vectors between different individuals as a disturbance term to guide individuals to undergo mutation updates, augment the richness and diversity of search agents, escape local optimal traps, continuously explore a broader solution space, and prominently intensify global search directionality and overall efficiency. For $f_{16}$, the optimal values, worst values, and mean values of the MSBKA, HBO, MOA, CPO, DRA, SOO, EAO, HEOA, SFOA, TGCOA, H5N1, MShOA and BKA are strictly equal to the global theoretical optimal solution; the standard deviations of the MSBKA and other mainstream comparison algorithms are zero. The MSBKA demonstrates the collaborative comprehensive advantages of the ranking-based differential mutation, simplex method, and elite opposition-based learning strategy to construct an integrated search mechanism of detection and mining escape, which approximates the theoretical optimal solution of all indicators, validates powerful global detection and local mining, and realizes strong superiority and practicality. For $f_{18}$, $f_{19}$ and $f_{20}$, the quantitative assessment metrics of the MSBKA are superior to those of the HBO, MOA, CPO, DRA, SFOA, H5N1 and BKA but inferior to those of the SOO, EAO, HEOA, TGCOA and MShOA. The MSBKA adopts the elite opposition-based learning strategy to construct a reverse learning escape channel, abate dependence on the initial population, and ensure operational stability and availability. For $f_{21}$ and $f_{22}$, the optimal values, worst values, and mean values of the MSBKA, HBO, MOA, CPO, DRA, SOO, SFOA, TGCOA, H5N1 and BKA are all consistent theoretical optimal solutions; the standard deviations of the MSBKA, HBO, MOA, SOO, SFOA, TGCOA, H5N1 and BKA are superior to those of the CPO, DRA, EAO, HEOA and MshOA. The MABKA manipulates the reflection, expansion, and contraction of the geometric transformation mechanisms to conduct a comprehensive and refined exploitation of the local area around the current elite search agent, accurately capture the local exact solution, approach the theoretical optimal solution, guide the population towards local optimum, diminish convergence oscillations and fluctuations, and strengthen convergence efficiency and solution quality. For $f_{23}$, the quantitative assessment metrics of the MSBKA, DRA, SOO, EAO, SFOA, TGCOA, H5N1 and MshOA are completely equivalent and converge accurately to the theoretical absolute optimal solution, strictly constructing a four-dimensional consistent solution set distribution. The optimal value, worst value, mean value, and standard deviation of the MSBKA are superior to those of the HBO, MOA, CPO, HEOA and BKA. The MSBKA materializes zero fluctuation and zero deviation deterministic optimization, realizes high uniformity of central tendency and dispersion degree, eliminates random interference, and demonstrates absolute convergence stability and solution superiority.

### 4.5. Convergence Analysis

**Figure 6:**
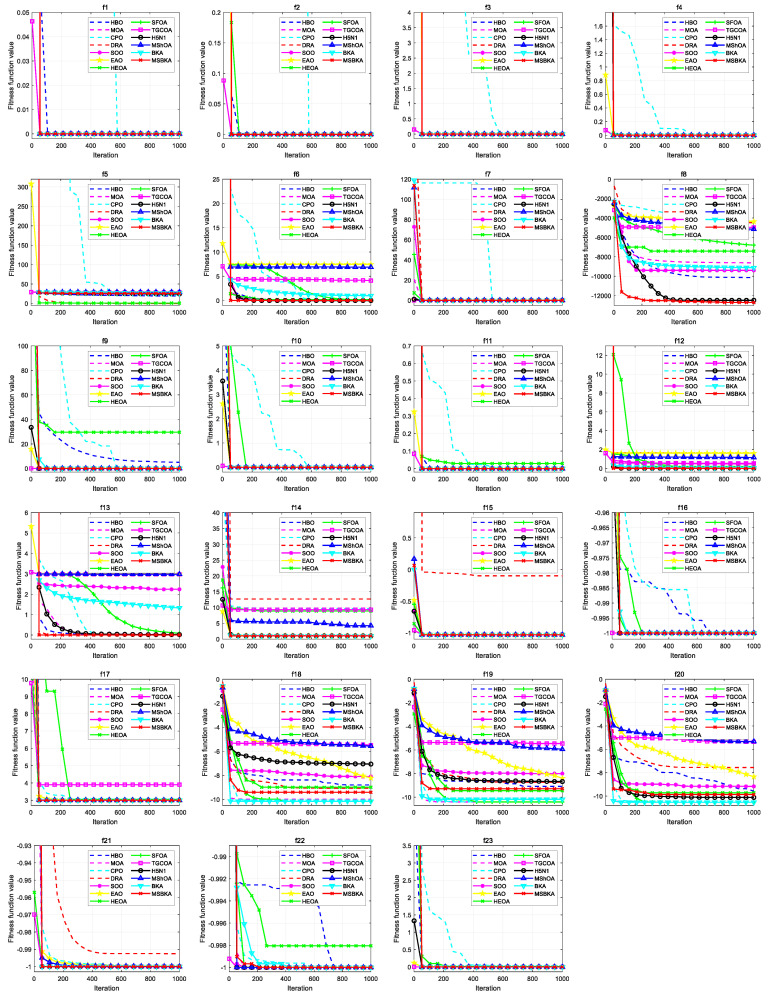
Convergence curves of MSBKA and mainstream algorithms on benchmark functions.

Figure 6 characterizes the convergence curves of MSBKA and mainstream algorithms on benchmark functions. For unimodal functions $f_{1}-f_{7}$, the most multidimensional quantitative criteria, productive detection efficiency, and exquisite mining precision of the MSBKA are substantially superior to those of the HBO, MOA, CPO, DRA, SOO, EAO, HEOA, SFOA, TGCOA, H5N1, MShOA and BKA. The MSBKA constructs a collaborative complementary optimization framework of a high-quality starting point, high-efficiency exploration and high-precision mining to overcome low population quality, scarcity information interaction and mindless migration following. For multimodal functions $f_{8}-f_{13}$, the most quantitative assessment metrics of the MSBKA are dramatically superior to those of the HBO, MOA, CPO, DRA, SOO, EAO, HEOA, SFOA, TGCOA, H5N1, MShOA and BKA. The MSBKA employs rationalized configuration and collaborative complementarity to accurately compensate for the pivotal weaknesses of the ponderous convergence speed, inadequate calculation accuracy, serious dimension disaster, negative diversity maintenance, strong parameter sensitivity, restricted initial population quality, insufficient robustness and stability, and inferior detection and mining. The MSBKA adopts elite reverse starting, differential mutation exploration and simplex method correction to heighten the solution quality and convergence efficiency, strengthen population interaction and alleviate premature convergence, facilitate efficient population migration and directional optimization of the worst individual, and intensify the robustness and stability. For fixed-dimensional multimodal functions $f_{14}-f_{23}$, the most multidimensional quantitative criteria, productive detection efficiency, and exquisite mining precision of MSBKA are prominently superior to those of the HBO, MOA, CPO, DRA, SOO, EAO, HEOA, SFOA, TGCOA, H5N1, MShOA and BKA in quantitative assessment metrics. The MSBKA manipulates deep exploitation, breadth exploration and direction guidance to comprehensively equalize global detection and local mining and systematically improve extraordinary stability and experimental repeatability.

### 4.6. Boxplot Analysis

**Figure 7:**
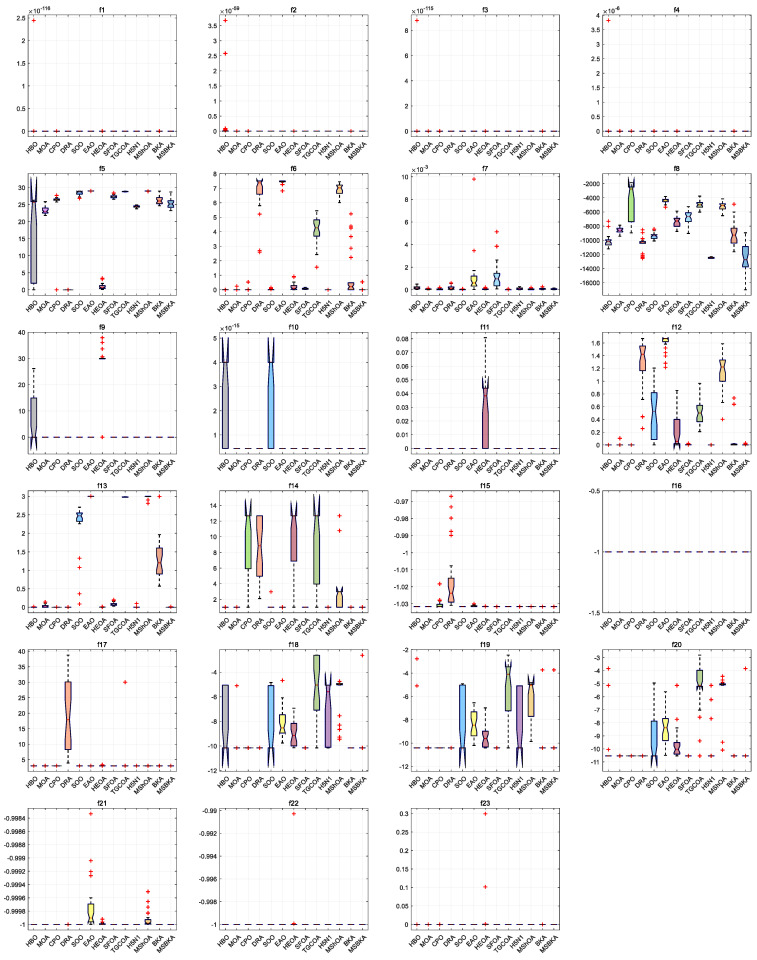
Boxplots of MSBKA and mainstream algorithms on benchmark functions.

Figure 7 characterizes the boxplots of MSBKA and mainstream algorithms on benchmark functions. The standard deviation is the square root of the variance and quantifies the dispersion or volatility between the data and the mean; it estimates the repeatability and reliability of algorithm models, measures stability and robustness, and identifies large fluctuations and outliers. For a small standard deviation with high consistency and strong stability, the data points are tightly clustered around the mean. For a large standard deviation with strong volatility and low stability, the data points are very scattered. For unimodal functions $f_{1}-f_{7}$, the most multidimensional quantitative standard deviation, convergence stability and discrete volatility of the MSBKA are dramatically superior to those of the HBO, MOA, CPO, DRA, SOO, EAO, HEOA, SFOA, TGCOA, H5N1, MShOA and BKA. The MSBKA adopts the ranking-based differential mutation to achieve dynamic parameter adjustment and uniform population distribution, restrain stochastic fluctuation, promote individual information exchange and maintain population diversity. The MSBKA adopts the simplex method to achieve stable search, accelerate local convergence, and reduce the oscillation and fluctuation of the optimal solution. The MSBKA adopts the elite opposition-based learning strategy to inject population diversity, reset search direction, avoid homogenization optimization, and strengthen the overall quality of the solution. For multimodal functions $f_{8}-f_{13}$, the MSBKA demonstrates noticeable superiority and robustness in terms of standard deviation, convergence stability and discrete volatility as compared to the HBO, MOA, CPO, DRA, SOO, EAO, HEOA, SFOA, TGCOA, H5N1, MShOA and BKA. The MSBKA demonstrates reasonable stability and repeatability to comprehensively optimize standard deviation and efficiently materialize low dispersion, excellent anti-interference, extensive adaptability and diminutive volatility. For fixed-dimensional multimodal functions $f_{14}-f_{23}$, the most multidimensional quantitative standard deviation, convergence stability and discrete volatility of the MSBKA are conspicuous lower than HBO, MOA, CPO, DRA, SOO, EAO, HEOA, SFOA, TGCOA, H5N1, MShOA and BKA. The ranking-based differential mutation can fundamentally compress result dispersion, refine the population structure, diminish the standard deviation to eliminate low-quality redundant individuals, reduce disorderly fluctuations, weaken ineffective fluctuation, standardize population evolution direction, and heighten consistency and superiority. The simplex method can intensify the stability of standard deviation, standardize the iterative logic to actualize refined local fine-tuning, reduce result oscillation, smooth the convergence process, and stabilize convergence objective. The elite opposition-based learning strategy can restrain extreme standard deviation, optimize the standard deviation distribution to promote the initial population quality, strengthen a stable foundation, enrich population diversity, and optimize the adaptation scenarios.

### 4.7. Wilcoxon Rank-Sum Test

The Wilcoxon rank-sum test, without the experimental data cautiously complying with the normal distribution and particular distribution assumption, is employed to validate whether there is a conspicuous discrepancy between two paired samples of the MSBKA and mainstream comparative algorithms [35]. The fundamental objective is to quantify accidental fluctuation, evade distribution assumption, intensify reliability and universality, eschew subjective speculation, and promote scientificity and credibility [36]. N/A represents “not applicable”. p≥0.05 authenticates that the performance discrepancy between the MSBKA and other algorithms has not yet attained a noticeable level; the search fluctuation is not sufficient to constitute a fundamental discrepancy. The MSBKA and mainstream comparative algorithms demonstrate the same competitiveness and integration to maintain the same level. p<0.05 not only authenticates the conspicuous and essential discrepancy between the MSBKA and other algorithms, but also demonstrates statistical availability and superiority and eliminates contingency and randomness [37]. The MSBKA exhibits strong competitiveness and effectiveness to strengthen the multidimensional quantitative criteria, detection efficiency and mining accuracy in quantitative assessment metrics. Table 3 emphasizes the numerical statistics results of MSBKA and mainstream algorithms of the Wilcoxon rank-sum test.

### 4.8. Simulation and Experimental Analysis

This section employs the ablation simulation to quantify the individual contributions of the ranking-based differential mutation, simplex method and elite opposition-based learning strategy to MSBKA’s gains rather than assuming them to be synergistic, which not only ensures the rigorousness, interpretability and significance of the research algorithm but also clarifies the quantifiability, traceability, and reproducibility of each strategy. The MSBKA is compared with the RBKA (BKA with ranking-based differential mutation), SBKA (BKA with simplex method), EBKA (BKA with elite opposition-based learning strategy), and BKA to verify the effectiveness and feasibility.

Table 4 emphasizes the numerical results of MSBKA, RBKA, SBKA, EBKA, BKA on benchmark functions. For all algorithms, the population size is N=50, the maximum iteration is T=1000, and independent reproduction is R=30. The optimal value, worst value, mean value and standard deviation are used as quantitative assessment metrics to achieve complementary advantages and collaborative search from the central tendency, dispersion degree and boundary performance, which comprehensively validates the excellence and utilization. The optimal value is employed to estimate the algorithm’s solution accuracy and to examine the global optimum, which reflects the global superficial detection and the localized refined mining. The worst value is employed to investigate the algorithm’s robustness and anti-interference ability, which reflects the synergistic complementarity of different strategies to avert local optima and ameliorate extreme output capability. The mean value is employed to quantify the algorithm’s overall effectiveness and average optimization performance, which reflects the synergy of strategies to enhance the comprehensive optimization performance. Standard deviation is employed to assess the algorithm’s stability and output consistency, which reflects population sorting optimization, local refined solution and fluctuation control. For unimodal functions $f_{1}-f_{7}$, the quantitative assessment metrics of the MSBKA converge to the global exact solution without any random fluctuations for $f_{1}$, $f_{2}$, $f_{3}$ and $f_{4}$. The quantitative assessment metrics of the MSBKA are superior to those of the RBKA, SBKA, EBKA and BKA; the MSBKA can extensively explore the solution space, accurately mine the optimal area, and achieve adaptive and coordinated control throughout the entire search process. For $f_{5}$, $f_{6}$ and $f_{7}$, the quantitative assessment metrics of the MSBKA are significantly superior to those of the RBKA, SBKA and BKA but slightly inferior to the EBKA; the MSBKA exhibits strong convergence consistency and collaborative complementarity to actualize directional exploration, facilitate exploitation efficiency, and ameliorate solution precision. For multimodal functions $f_{8}-f_{13}$, the optimal values, worst values, mean values, and standard deviations of the MSBKA, RBKA, SBKA, EBKA and BKA maintain the same numerical statistical results and precisely effectuate the global exact extremum solutions with zero fluctuation for $f_{9}$, $f_{10}$ and $f_{11}$. These algorithms exhibit strong practicability and reliability to materialize theoretically optimal convergence consistency and consummate statistical robustness. The quantitative assessment metrics of MSBKA significantly outperform those of the RBKA, SBKA, EBKA and BKA for $f_{8}$, $f_{12}$ and $f_{13}$; the MSBKA collaboratively achieves globalized discovery and localized extraction to restrain some deficiencies of the ponderous convergence speed, inadequate calculation accuracy, easy search stagnation, excessive parameter sensitivity, insufficient population diversity, restricted initial population quality, disorganized global detection, and local extraction. For fixed-dimensional multimodal functions $f_{14}-f_{23}$, the optimal values, worst values, and mean values of the MSBKA, RBKA, SBKA, EBKA and BKA maintain the equivalent measurement level and exhibit a theoretically optimal deterministic convergence solution; for $f_{15}$, $f_{16}$ $f_{17}$, $f_{21}$ and $f_{22}$, the standard deviations of the MSBKA and other mainstream comparison algorithms maintain the same order of magnitude. The MSBKA exhibits extraordinary fault tolerance and versatility to imitate population competition and collaboration, dynamically guide search direction, maintain population multiplicity, intensify solution quality, and achieve multi-dimensional superiority. For $f_{18}$, $f_{19}$ and $f_{20}$, the optimal values, worst values, mean values and standard deviations of the MSBKA are slightly inferior to those of the RBKA, SBKA, EBKA and BKA; the MSBKA demonstrates the global exploration and local exploitation of turbulent fluctuations, decentralized stability and search uncontrollability. For $f_{14}$ and $f_{23}$, the quantitative assessment metrics of the MSBKA are superior to those of the RBKA, SBKA, EBKA and BKA; the MSBKA manipulates deep exploitation and breadth exploration to materialize the rationalized configuration between different strategies, diminish homogenization competition, eschew local optimal traps, intensify information interaction and migration, facilitate population directional exploitation, ameliorate calculation precision, and consolidate stability and practicality.

**Figure 8:**
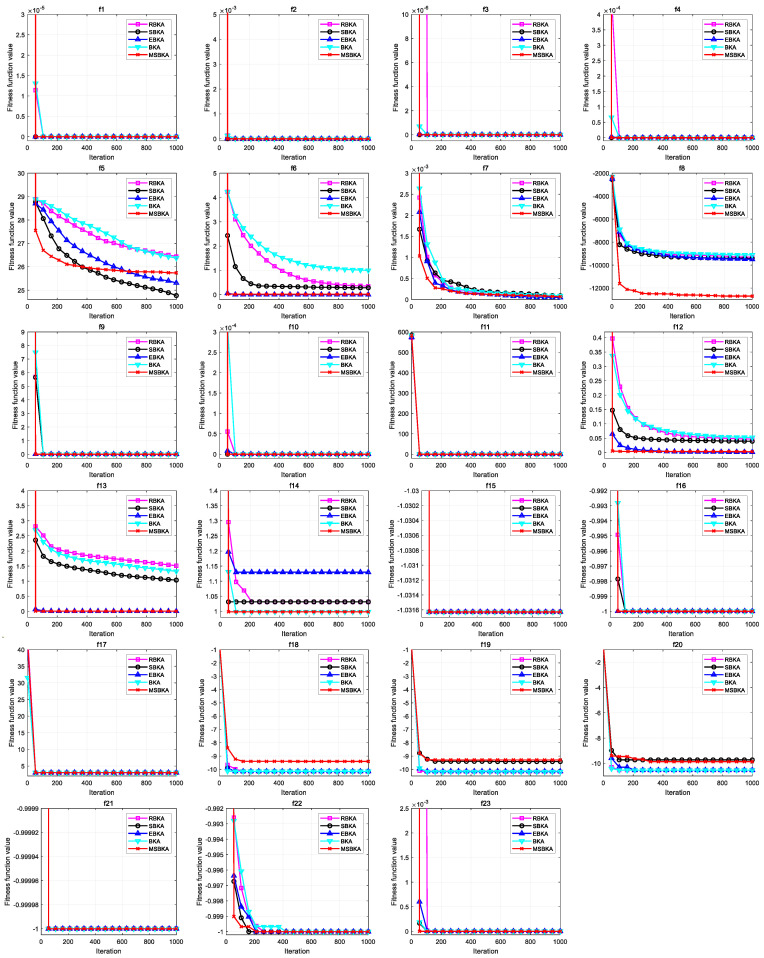
Convergence curves of MSBKA, RBKA, SBKA, EBKA, BKA on benchmark functions.

Figure 8 characterizes the convergence curves of MSBKA, RBKA, SBKA, EBKA, and BKA on benchmark functions. For unimodal functions $f_{1}-f_{7}$, the most quantitative assessment metrics, detection efficiency, and mining precision of the MSBKA are substantially superior to those of the RBKA, SBKA, EBKA and BKA. The MSBKA exhibits strong superiority and reliability to evaluate individual fitness differences and differential information, promote population information exchange, maintain overall search efficiency, regulate directional search, ensure global detection accuracy, clarify search directionality, and reduce ineffective detection. For multimodal functions $f_{8}-f_{13}$, the most optimal values, worst values, mean values, standard deviations, exploration speed and exploitation accuracy of the MSBKA are dramatically superior to those of the RBKA, SBKA, EBKA and BKA. The MSBKA exhibits strong robustness and practicality to evaluate individual fitness differences and differential information, promote population information exchange, maintain overall search efficiency, regulate directional search, ensure global detection accuracy, clarify search directionality, and reduce ineffective detection. For fixed-dimensional multimodal functions $f_{14}-f_{23}$, the most quantitative assessment metrics, convergence rate, and computational accuracy of the MSBKA are prominently superior to those of the RBKA, SBKA, EBKA and BKA in terms of the most optimal values, worst values, mean values, and standard deviations. The MSBKA exhibits strong stability and repeatability to evaluate individual fitness differences and differential information, promote population information exchange, maintain overall search efficiency, regulate directional search, ensure global detection accuracy, clarify search directionality, and reduce ineffective detection. The MSBKA demonstrates strong superiority and robustness to equalize global detection and local mining and achieve comprehensive improvement of convergence speed and computational precision.

**Figure 9:**
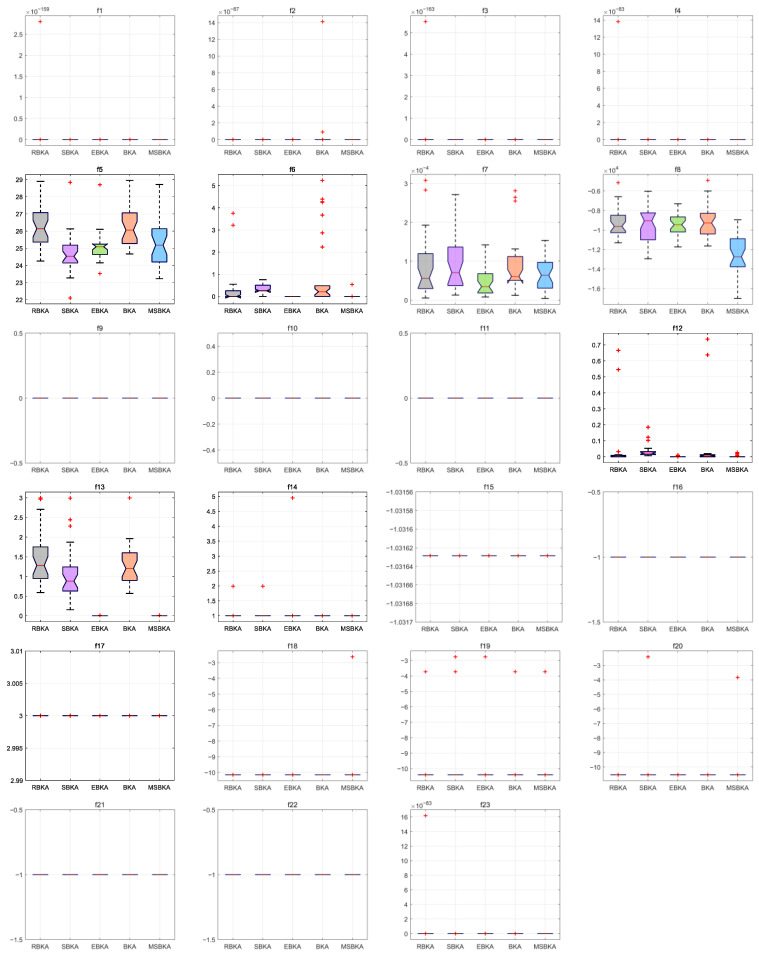
Boxplots of MSBKA, RBKA, SBKA, EBKA, and BKA on benchmark functions.

Figure 9 characterizes the boxplots of the MSBKA, RBKA, SBKA, EBKA, and BKA on benchmark functions. For unimodal functions $f_{1}-f_{7}$, the most multidimensional quantitative standard deviation, convergence consistency, discrete volatility, dimensional adaptability and output repeatability of the MSBKA are dramatically superior to those of the RBKA, SBKA, EBKA and BKA. The MSBKA demonstrates distinguished comprehensive superiority and collaborative integration to prominently diminish standard deviation, materialize convergence stability and fluctuation control of solutions, and dynamically equalize global detection and local mining. For multimodal functions $f_{8}-f_{13}$, the MSBKA demonstrates noticeable exploration stability and exploitation robustness in terms of the most multidimensional quantitative standard deviation, convergence consistency, discrete volatility, dimensional adaptability and output repeatability when the MSBKA is compared to the RBKA, SBKA, EBKA and BKA. The MSBKA integrates the ranking-based differential mutation, simplex method and elite opposition-based learning strategy to constrain iterative fluctuation synergistically, accurately maintain an extremely low level of standard deviation, resist the disturbance interference of high-dimensional redundancy and low-quality individuals, search for deviation compensation, initialize population randomness, sustain population stability, standardize the convergence process, and consolidate stability and practicality. For fixed-dimensional multimodal functions $f_{14}-f_{23}$, the most multidimensional quantitative standard deviation, convergence consistency, discrete volatility, dimensional adaptability and output repeatability of the MSBKA are conspicuously lower than the RBKA, SBKA, EBKA and BKA. The MSBKA not only employs the ranking-based differential mutation, simplex method and elite opposition-based learning strategy to effectively overcome the shortcomings of the large standard deviation and insufficient stability of the BKA and mainstream comparison algorithms but also actualizes the collaborative enhancement of the standard deviation, calculation accuracy, and convergence speed, stability and practicality.

Table 5 emphasizes the numerical statistics results of the MSBKA and mainstream algorithms in the Wilcoxon rank-sum test. The MSBKA exhibits strong practicality and responsibility to expedite convergence efficiency, ameliorate solution precision, and reinforce stability and robustness. The experimental results authenticate the conspicuous and essential discrepancy between the MSBKA and other algorithms.

## 5. MSBKA for Accomplishing Constrained Engineering Applications

The MSBKA is employed to accomplish the constrained engineering applications of the three-bar truss [38], tension/compression spring [39], gear train [40], car side impact [41], multiple-disk clutch brake [42], and rolling element bearing [43]. The principal mission is to construct a robust constraint search mechanism to equalize exploration breadth and exploitation depth, as well as stably and accurately approximate the globally feasible optimal solution in terms of initial solution blindness, unbalanced global detection and local mining, and weak constraint boundary search ability. The MSBKA can directly materialize the most cost-effective and standardized design parameters, dramatically diminish engineering trial and error costs, and prominently promote the economic reliability and adaptive robustness.

### 5.1. Three-Bar Truss

**Figure 10:**
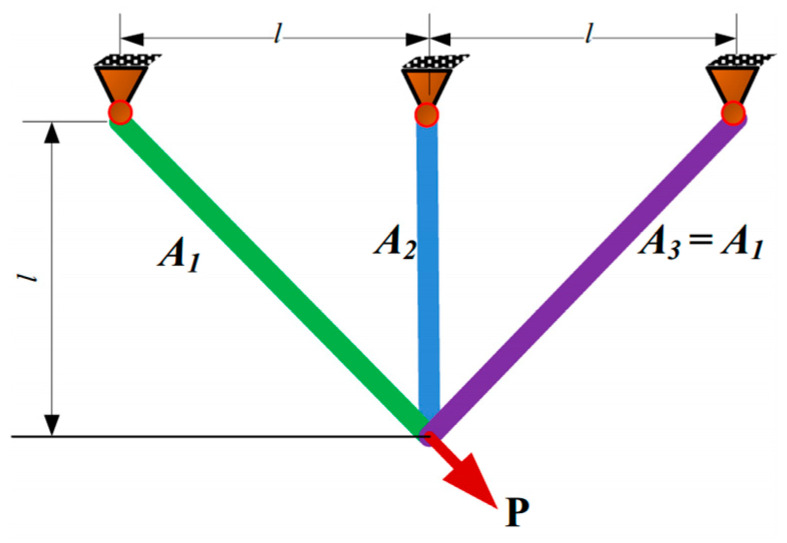
Three-bar truss.

The principal mission is to retrench the optimum counterweight as characterized in Figure 10, which encompasses multidimensional quantitative elements: double transections $A_{1}$ and $A_{2}$. The intrinsic quantitative logic of the mathematical framework is decomposed as follows:

Consider

(21) $$x=[x_{1}\;x_{2}]=[A_{1}\;A_{2}]$$

Minimize

(22) $$f(x)=(2\sqrt{2}x_{1}+x_{2})\times l$$

Subject to

(23) $$g_{1}(x)=\frac{\sqrt{2}x_{1}+x_{2}}{\sqrt{2}x_{1}^{2}+2x_{1}x_{2}}P-\sigma \leq 0$$

(24) $$g_{2}(x)=\frac{x_{2}}{\sqrt{2}x_{2}+2x_{1}x_{2}}P-\sigma \leq 0$$

(25) $$g_{3}(x)=\frac{1}{\sqrt{2}x_{2}+x_{1}}P-\sigma \leq 0$$

(26) $$l=100\;\mathrm{cm},\;P=2\;\mathrm{kN}/{\mathrm{cm}}^{2},\;\sigma =2\;\mathrm{kN}/{\mathrm{cm}}^{2}$$

Variable range

(27) $$0\leq x_{1},x_{2}\leq 1$$

Table 6 emphasizes the numerical statistics results of the three-bar truss. The MSBKA cultivates the high-precision and high-efficiency incomparable counterweight f(x)=263.8745 with comprehensive, distinguished moderation and quantification elements x=0.765579, 0.468012. The MSBKA employs differential quantization and sorting selection to accomplish dynamic regulation of global detection and local mining, eliminate redundant and inferior individuals, materialize high-quality gene inheritance and population diversity evolution, diminish ineffective search and operational fluctuation, and promote search stability and iteration efficiency.

### 5.2. Tension/Compression Spring

**Figure 11:**
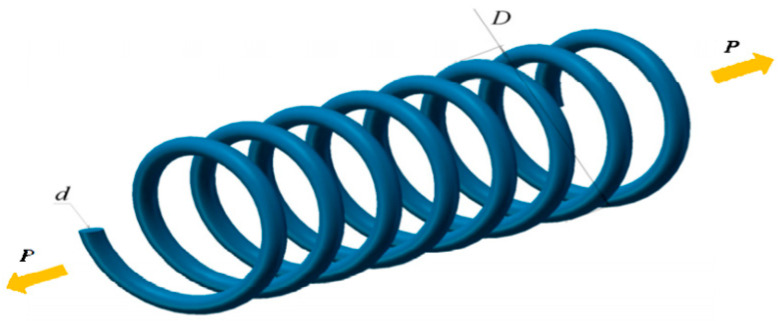
Tension/compression spring.

The principal mission is to retrench the optimum counterweight as characterized in Figure 11, which encompasses multidimensional quantitative elements: thickness of the spring wire d, average helix diameter D, and coils participating in deformation N. The intrinsic quantitative logic of the mathematical framework is decomposed as follows:

Consider

(28) $$x=[x_{1}\;x_{2}\;x_{3}\;]=[d\;D\;P]\;$$

Minimize

(29) $$f(x)=(x_{3}+2)x_{2}x_{1}^{2}$$

Subject to

(30) $$g_{1}(x)=1-\frac{x_{2}^{3}x_{3}}{71785x_{1}^{4}}\leq 0$$

(31) $$g_{2}(x)=\frac{4x_{2}^{2}-x_{1}x_{2}}{12566(x_{2}x_{1}^{3}-x_{1}^{4})}+\frac{1}{5108x_{1}^{2}}\leq 0$$

(32) $$g_{3}(x)=1-\frac{140.45x_{1}}{x_{2}^{2}x_{3}}\leq 0$$

(33) $$g_{4}(x)=\frac{x_{1}+x_{2}}{1.5}-1\leq 0$$

Variable range

(34) $$0.05\leq x_{1}\leq 2,\;0.25\leq x_{2}\leq 1.3,\;2\leq x_{3}\leq 15$$

Table 7 emphasizes the numerical statistics results of the tension/compression spring. The MSBKA cultivates the high-precision and high-efficiency irreplaceable counterweight f(x)=0.012478 with the comprehensive outstanding moderation and quantification elements x=0.051785,0.353691,11.143834. The MSBKA utilizes the reflection, expansion, and contraction of the simplex method to construct a momentous search structure of the elite individuals, materialize refinement and controllability of local mining, eliminate random perturbation and oscillation, explicitly search logicality and directionality, strengthen practicality and repeatability, and accurately approximate the global high-quality solution.

### 5.3. Gear Train

**Figure 12:**
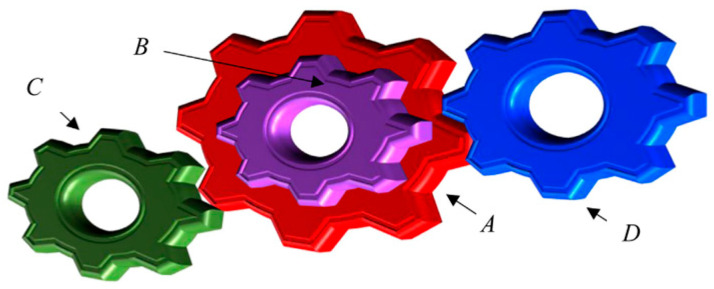
Gear train.

The principal mission is to retrench the optimum cost-effectiveness as characterized in Figure 12, which encompasses multidimensional quantitative elements: respective tooth counts for gears $n_{A}$, $n_{B}$, $n_{C}$ and $n_{D}$. The intrinsic quantitative logic of the mathematical framework is decomposed as follows:

Consider

(35) $$x=[x_{1}\;x_{2}\;x_{3}\;x_{4}]=[n_{A}\;n_{B}\;n_{C}\;n_{D}]$$

Minimize

(36) $$f(x)={\left(\frac{1}{6.931}-\frac{x_{3}x_{2}}{x_{1}x_{4}}\right)}^{2}$$

Variable range

(37) $$12\leq x_{i}\leq 60,\;i=1,2,\ldots ,4$$

Table 8 emphasizes the numerical statistics results of the gear train. The MSBKA cultivates the high-precision and high-efficiency incomparable cost-effectiveness $f(x)=1.7869\times {\mathrm{10}}^{-19}$ with the comprehensive exceptional moderation and quantification elements x=56,18,20,45. The MSBKA operates the elite opposition-based learning strategy to actualize the dynamic equilibrium between contraction and expansion, furnish a non-abrupt large step transition, strengthen serviceability and versatility, broaden discovery scope and population heterogeneity, and strengthen convergence efficiency and solution optimality.

### 5.4. Car Side Impact

**Figure 13:**
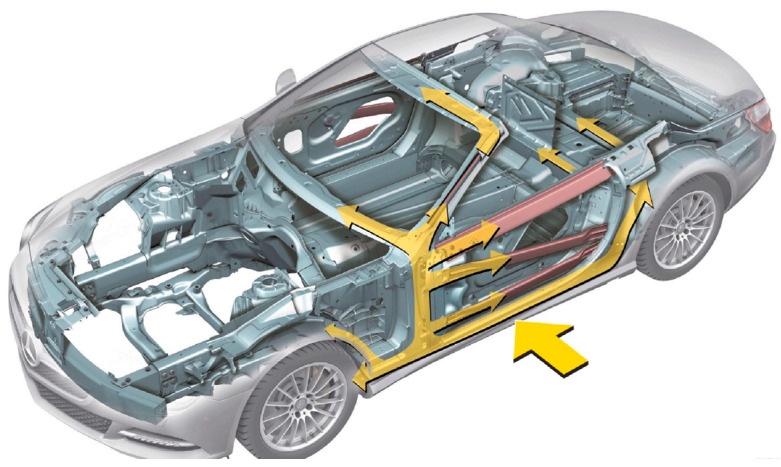
Car side impact.

The principal mission is to retrench the optimum counterweight as characterized in Figure 13, which includes multidimensional quantitative elements: thickness specifications of B-pillar inner panel $x_{1}$, B-pillar reinforcement $x_{2}$, floor side inner panel $x_{3}$, cross members $x_{4}$, door beam $x_{5}$, door beltline reinforcement $x_{6}$, roof rail $x_{7}$, B-pillar inner panel $x_{8}$, floor side inner panel $x_{9}$, barrier height $x_{10}$, and impact location $x_{11}$. The intrinsic quantitative logic of the mathematical framework is decomposed as follows:

Consider

(38) $$x=[x_{1}\;x_{2}\;x_{3}\;x_{4}\;x_{5}\;x_{6}\;x_{7}\;x_{8}\;x_{9}\;x_{10}\;x_{11}]$$

Minimize

(39) $$f(x)=1.98+4.90x_{1}+6.67x_{2}+6.98x_{3}+4.01x_{4}+1.78x_{5}+2.73x_{7}$$

Subject to

(40) $$\begin{array}{lll} g_{1}(x) & = & 1.16-0.3717x_{2}x_{4}-0.00931x_{2}x_{10}-0.484x_{3}x_{9}\\ & & +0.01343x_{6}x_{10}\leq 1 \end{array}$$

(41) $$\begin{array}{lll} g_{2}(x) & = & 0.261-0.0159x_{1}x_{2}-0.188x_{1}x_{8}-0.019x_{2}x_{7}\\ & & +0.0144x_{3}x_{5}+0.0008757x_{5}x_{10}+0.080405x_{6}x_{9}\\ & & +0.00139x_{8}x_{11}+0.00001575x_{10}x_{11}\leq 0.32 \end{array}$$

(42) $$\begin{array}{lll} g_{3}(x) & = & 0.214+0.00817x_{5}-0.131x_{1}x_{8}-0.0704x_{1}x_{9}\\ & & +0.03099x_{2}x_{6}-0.018x_{2}x_{7}+0.0208x_{3}x_{8}+0.121x_{3}x_{9}\\ & & -0.00364x_{5}x_{6}+0.0007715x_{5}x_{10}-0.000535x_{6}x_{10}\\ & & +0.00121x_{8}x_{11}\leq 0.32 \end{array}$$

(43) $$\begin{array}{lll} g_{4}(x) & = & 0.074-0.061x_{2}-0.163x_{3}x_{8}+0.001232x_{3}x_{10}\\ & & -0.166x_{7}x_{9}+0.227x_{2}^{2}\leq 0.32 \end{array}$$

(44) $$\begin{array}{lll} g_{5}(x) & = & 28.98+3.818x_{3}-4.2x_{1}x_{2}+0.0207x_{5}x_{10}+6.63x_{6}x_{9}\\ & & -7.7x_{7}x_{8}+0.32x_{9}x_{10}\leq 32 \end{array}$$

(45) $$\begin{array}{lll} g_{6}(x) & = & 33.86+2.95x_{3}+0.1792x_{10}-5.057x_{1}x_{2}-11.0x_{2}x_{8}\\ & & -0.0215x_{5}x_{10}-9.98x_{7}x_{8}+22.0x_{8}x_{9}\leq 32 \end{array}$$

(46) $$g_{7}(x)=46.36-9.9x_{2}-12.9x_{1}x_{8}+0.1107x_{3}x_{10}\leq 32$$

(47) $$\begin{array}{lll} g_{8}(x) & = & 4.72-0.5x_{4}-0.19x_{2}x_{3}-0.0122x_{4}x_{10}\\ & & +0.009325x_{6}x_{10}+0.000191x_{11}^{2}\leq 4 \end{array}$$

(48) $$\begin{array}{lll} g_{9}(x) & = & 10.58-0.674x_{1}x_{2}-1.95x_{2}x_{8}+0.02054x_{3}x_{10}\\ & & -0.0198x_{4}x_{10}+0.028x_{6}x_{10}\leq 9.9 \end{array}$$

(49) $$\begin{array}{lll} g_{10}(x) & = & 16.45-0.489x_{3}x_{7}-0.843x_{5}x_{6}+0.0432x_{9}x_{10}\\ & & -0.0556x_{9}x_{11}-0.000786x_{11}^{2}\leq 15.7 \end{array}$$

Variable range

(50) $$0.5\leq x_{1}-x_{7}\leq 1.5,\;x_{8},x_{9}\in (0.192,0.345),\;-30\leq x_{10},x_{11}\leq 30$$

Table 9 emphasizes the numerical statistics results of the car side impact. The MSBKA cultivates the high-precision and high-efficiency irreplaceable counterweight f(x)=22.88942 with the comprehensive transcendent moderation and quantification elements x=0.5, 1.11674, 0.5, 1.31276, 0.5, 1.5, 0.5, 0.34992, 0.191, −19.53974, −0.02683. The MSBKA materializes the synergistic complementarity of the ranking-based differential mutation, simplex method and elite opposition-based learning strategy to diminish homogenization competition, reconcile global detection and local mining, abate parameter sensitivity, eschew local optimal traps, guide intelligent search direction, strengthen information interaction, and ameliorate calculation precision.

### 5.5. Multiple-Disk Clutch Brake

**Figure 14:**
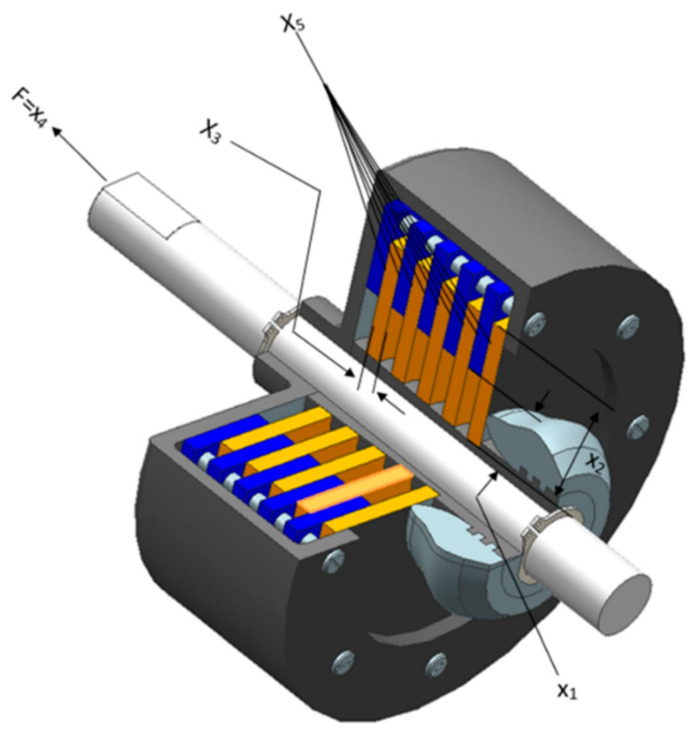
Multiple-disk clutch brake.

The principal mission is to retrench the optimum counterweight as characterized in Figure 14, which encompasses multidimensional quantitative elements: disk thickness t, inner radius $r_{i}$, outer radius $r_{o}$, actuating force F, and the number of friction surfaces Z. The intrinsic quantitative logic of the mathematical framework is decomposed as follows:

Consider

(51) $$x=[x_{1}\;x_{2}\;x_{3}\;x_{4}\;x_{5}]=[r_{i}\;r_{0}\;t\;F\;Z]$$

Minimize

(52) $$f(x)=\pi t\rho (r_{0}^{2}-r_{i}^{2})(Z+1)$$

Subject to

(53) $$g_{1}(x)=r_{0}-r_{i}-\Delta r\geq 0$$

(54) $$g_{2}(x)=l_{\max}-(Z+1)(t+\delta )\geq 0$$

(55) $$g_{3}(x)=p_{\max}+p_{rz}\geq 0$$

(56) $$g_{4}(x)=p_{\max}v_{sr\;\max}-p_{rz}v_{sr}\geq 0$$

(57) $$g_{5}(x)=v_{sr\;\max}-v_{sr}\geq 0$$

(58) $$g_{6}(x)=T_{\max}-T\geq 0$$

(59) $$g_{7}(x)=M_{h}-sM_{s}\geq 0$$

(60) $$g_{8}(x)=T\geq 0$$

(61) $$M_{h}=\frac{2}{3}\mu FZ\frac{r_{0}^{3}-r_{i}^{3}}{r_{0}^{2}-r_{i}^{2}}$$

(62) $$p_{rz}=\frac{F}{\pi (r_{0}^{2}-r_{i}^{2})}$$

(63) $$v_{sr}=\frac{2\pi n(r_{0}^{3}-r_{i}^{3})}{90(r_{0}^{2}-r_{i}^{2})}$$

(64) $$T=\frac{I_{z}\pi n}{30(M_{h}+M_{f})}$$

(65) $$\Delta r=20\;\mathrm{mm},\;I_{z}=55\;{\mathrm{kgmm}}^{2},\;p_{\max}=1\;\mathrm{Mpa},\;F_{\max}=1000\;N$$

(66) $$T_{\max}=15\;s,\;\mu =0.5,\;s=1.5,\;M_{s}=40\;\mathrm{Nm}$$

(67) $$M_{f}=3\;\mathrm{Nm},\;n=250\;\mathrm{rpm}$$

(68) $$v_{sr\;\max}=10\;m/s,\;l_{\max}=30\;\mathrm{mm},\;r_{i\;\min}=60$$

(69) $$r_{i\;\max}=80,\;r_{o\;\min}=90$$

(70) $$r_{o\;\max}=110,\;t_{\min}=1.5,\;t_{\max}=3,\;F_{\min}=600$$

(71) $$F_{\max}=1000,\;Z_{\min}=2,\;Z_{\max}=9$$

Table 10 emphasizes the numerical statistics results of the multiple-disk clutch brake. The MSBKA cultivates the high-precision and high-efficiency incomparable counterweight f(x)=0.27642 with comprehensive extraordinary moderation and quantification elements x=70, 90, 1, 987, 2. The MSBKA demonstrates extraordinary fault tolerance and universality to materialize the globally accurate extremum solution, exhibit comprehensive consistency and scalability, construct high-quality solution space distribution, diminish convergence oscillation, validate powerful global detection and local mining, and ensure operational stability and availability.

### 5.6. Rolling Element Bearing

**Figure 15:**
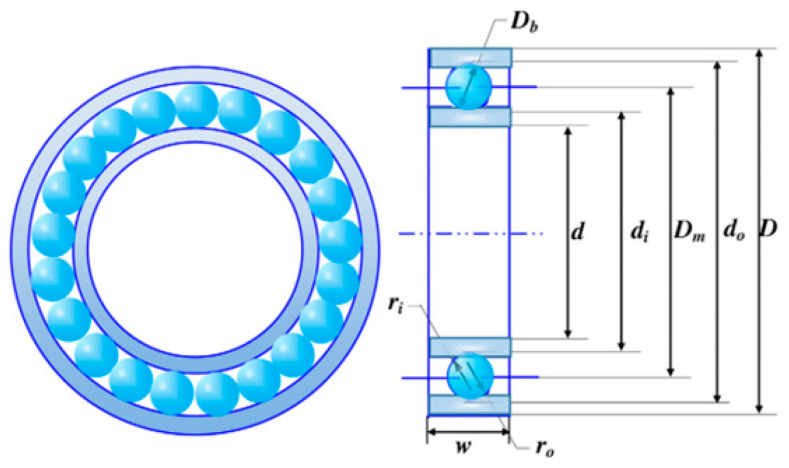
Rolling element bearing.

The principal mission is to retrench the optimum cost-effectiveness as characterized in Figure 15, which encompasses multidimensional quantitative elements: pitch diameter $D_{m}$, ball diameter $D_{b}$, number of balls Z, inner ring $f_{i}$, outer ring $f_{o}$, and raceway curvature coefficients $K_{D\min}$, $K_{D\max}$, ε, e, ζ. The intrinsic quantitative logic of the mathematical framework is decomposed as follows:

Consider

(72) $$\begin{array}{l} x=[x_{1}\;x_{2}\;x_{3}\;x_{4}\;x_{5}\;x_{6}\;x_{7}\;x_{8}\;x_{9}\;x_{10}]\\ \;=[D_{m}\;D_{b}\;Z\;f_{i}\;f_{o}\;K_{D\min}\;K_{D\max}\;\varepsilon \;e\;\zeta ] \end{array}$$

Minimize

(73) $$C_{d}=\left\{\begin{array}{ll} f_{c}Z^{2/3}D_{b}^{1.8}, & if\;D\leq 25.4\;\mathrm{mm}\\ 3.647f_{c}Z^{2/3}D_{b}^{1.4}, & if\;D>25.4\;\mathrm{mm} \end{array}\right.$$

Subject to

(74) $$g_{1}(x)=\frac{\phi_{0}}{2\sin^{-1}(D_{b}/D_{m})}-Z+1\leq 0$$

(75) $$g_{2}(x)=2D_{b}-K_{D\min}(D-d)\geq 0$$

(76) $$g_{3}(x)=K_{D\max}(D-d)-2D_{b}\geq 0$$

(77) $$g_{4}(x)=\zeta B_{\omega }-D_{b}\leq 0$$

(78) $$g_{5}(x)=D_{m}-0.5(D+d)\geq 0$$

(79) $$g_{6}(x)=(0.5+e)(D+d)-D_{m}\geq 0$$

(80) $$g_{7}(x)=0.5(D-D_{m}-D_{b})-\varepsilon D_{b}\geq 0$$

(81) $$g_{8}(x)=f_{i}\geq 0.515$$

(82) $$g_{9}(x)=f_{o}\geq 0.515$$

(83) fc=37.911+1.041−r1+r1.72fi(2fo−1)fo(2fi−1)0.41103−0.3×r0.3(1−r)1.39(1+r)1/32fi2fi−10.41

(84) $$x={\left\{\frac{\left(D-d\right)}{2}-3\frac{T}{4}\right\}}^{2}+{\left\{\frac{D}{2}-\frac{T}{4}-D_{b}\right\}}^{2}-{\left\{\frac{d}{2}+\frac{T}{4}\right\}}^{2}$$

(85) $$y=2\left\{\frac{\left(D-d\right)}{2}-3\frac{T}{4}\right\}\left\{\frac{D}{2}-\frac{T}{4}-D_{b}\right\}$$

(86) $$\phi_{o}=2\pi -2\cos^{-1}\left(\frac{x}{y}\right)$$

(87) $$r=\frac{D_{b}}{D_{m}},\;f_{i}=\frac{r_{i}}{D_{b}},\;f_{o}=\frac{r_{o}}{D_{b}},\;T=D-d-2D_{b}$$

(88) $$D=160,\;d=90,\;B_{\omega }=30,\;r_{i}=r_{o}=11.033$$

Variable range

(89) $$0.5(D+d)\leq D_{m}\leq 0.6(D+d)$$

(90) $$0.15(D-d)\leq D_{b}\leq 0.45(D-d)$$

(91) $$4\leq Z\leq 50,\;0.515\leq f_{i},f_{o}\leq 0.6$$

(92) $$0.4\leq K_{D\min}\leq 0.5,\;0.6\leq K_{D\min}\leq 0.7$$

(93) 0.3≤ε≤0.4, 0.02≤e≤0.1, 0.6≤ζ≤0.85

Table 11 emphasizes the numerical statistics results of the rolling element bearing. The MSBKA cultivates the high-precision and high-efficiency irreplaceable counterweight f(x)=85012.5432 with the comprehensive transcendental moderation and quantification elements x=125, 21.7648, 11.3921, 0.5151, 0.5151, 0.4, 0.7, 0.3, 0.02, 0.6. The MSBKA manipulates the rationalized configuration and collaborative complementarity of deep exploitation, breadth exploration and direction guidance to intensify information interaction and mindless migration following, facilitate efficient population migration and directional exploitation, reduce the oscillation and fluctuation of the optimal solution, and consolidate stability and practicality.

## 6. Conclusions and Future Research

This paper constructs the MSBKA based on the ranking-based differential mutation, simplex method and elite opposition-based learning strategy to resolve the benchmark functions and engineering designs. The purpose is to reveal unambiguous stratification and progression; quantify the comprehensive rationality of convergence speed, solution accuracy, global optimization ability, robustness and stability in function optimization; and demonstrate efficient detection and mining to verify the practicability and universality of dealing with nonlinear, multivariable, multi-constraint, nonconvex, high-dimensional coupling in engineering applications. The BKA with the Cauchy mutation strategy and the leader selection strategy imitates the high-altitude circling exploration, fixed-point diving attack and group cooperative migration of the black-winged kites to construct an iterative optimization mechanism and materialize efficient solutions. The BKA exhibits some serious deficiencies of inadequate population information, inferior detection and mining, ponderous convergence speed, inadequate calculation accuracy, fast diversity attenuation, insufficient robustness and stability, high parameter sensitivity, serious dimension disaster, and negative diversity maintenance. The ranking-based differential mutation can guide the population’s orderly evolution, diminish search blindness, elevate population information utilization, stabilize convergence efficiency, shorten the optimization period, and sustain population diversity. The simplex method can intensify local fine mining, ameliorate optimization accuracy, weaken parameter sensitivity, eschew mutual restriction between convergence efficiency and calculation accuracy, and resolve the complex optimization scenarios. The elite opposition-based learning strategy can eliminate population homogeneity, expand search space, promote robustness and adaptability, fortify the quality of initial and iterative solutions, restrain premature convergence, and maintain population diversity. The MSBKA manipulates multiple strategies, comprehensive optimization advantages and a collaborative complementary mechanism to actualize directional detection, deep mining and diversity maintenance. The MSBKA is compared with the HBO, MOA, CPO, DRA, SOO, EAO, HEOA, SFOA, TGCOA, H5N1, MShOA and BKA. The experimental results demonstrate that the MSBKA not only exhibits reinforced superiority and practicability to actualize complementary advantages among strategies and dynamic switches in the global detection and the local mining but also employs excellent adaptability and feasibility to materialize faster convergence efficiency, higher solution precision and stronger stability and robustness.

In future research, we will execute the systematic analysis from the following three perspectives: (1) We will leverage the resources of Anhui Provincial Engineering Research Center for Understory Crop Intelligent Equipment to upgrade autonomous decision-making and collaborative efficiency of intelligent equipment, actualize the path and harvest planning of the agricultural and forestry robot path planning, materialize multi-source perception sensor networks deployment, attain collaborative scheduling of equipment clusters, accomplish lightweight equipment structure, recognize equipment control parameter self-tuning, and promote the autonomy, adaptability, cooperativity and economy of intelligent equipment systems. (2) We will standardize the same maximum fitness evaluations to ensure a fair comparison of performance for different algorithms. We will further enhance the scientificity and adaptability of parameter determination while retaining the practicality and stability of existing algorithms and adapt to a wider range of optimization scenarios. We will conduct an in-depth analysis of the intrinsic correlation between parameter values and algorithm performance to clarify the theoretical basis for parameter robustness intervals and strengthen the continuity, strictness, completeness, and universality of the algorithm.

## Figures and Tables

**Table 1:** Benchmark functions.

Benchmark Functions	Dim	Range	$f_{\min}$
$f_{1}=\sum_{i=1}^{n}x_{i}^{2}$	30	[−100, 100]	0
$f_{2}(x)=\sum_{i=1}^{n}\left|x_{i}\right|+\prod_{i=1}^{n}\left|x_{i}\right|$	30	[−10, 10]	0
$f_{3}(x)=\sum_{i=1}^{n}{\left(\sum_{j=1}^{i}x_{j}\right)}^{2}$	30	[−100, 100]	0
$f_{4}(x)=\max_{i}\{|x_{i}|,1\leq i\leq n\}$	30	[−100, 100]	0
$f_{5}(x)=\sum_{i=1}^{n-1}\left[100{\left(x_{i+1}-x_{i}^{2}\right)}^{2}+{\left(x_{i}-1\right)}^{2}\right]$	30	[−30, 30]	0
$f_{6}(x)=\sum_{i=1}^{n}{\left([x_{i}+0.5]\right)}^{2}$	30	[−100, 100]	0
$f_{7}(x)=\sum_{i=1}^{n}ix_{i}^{4}+random[0,1)$	30	[−1.28, 1.28]	0
$f_{8}(x)=\sum_{i=1}^{n}\left(-x_{i}\sin(\sqrt{\left|x_{i}\right|}\right))$	30	[−500, 500]	−12,569.5
$f_{9}(x)=\sum_{i=1}^{n}\left[x_{i}^{2}-10\cos(2\pi x_{i})+10\right]$	30	[−5.12, 5.12]	0
$\begin{array}{l} f_{10}(x)=-20\exp\left(-0.2\sqrt{\frac{1}{n}\sum_{i=1}^{n}x_{i}^{2}}-\exp\left(\frac{1}{n}\sum_{i=1}^{n}\cos2\pi x_{i}\right)\right)\\ +20+e \end{array}$	30	[−32, 32]	0
$f_{11}(x)=\frac{1}{4000}\sum_{i=1}^{n}\left(x_{i}^{2}\right)-\prod_{i=1}^{n}\cos\left(\frac{x_{i}}{\sqrt{i}}\right)+1$	30	[−600, 600]	0
$\begin{array}{l} f_{12}(x)=\frac{\pi }{n}\left\{10\sin^{2}(\pi y_{1})+\sum_{i=1}^{n-1}\begin{array}{l} {\left(y-1\right)}^{2}[1+10\sin^{2}(\pi y_{1})]\\ +{\left(y_{n}-1\right)}^{2} \end{array}\right\}\\ +\sum_{i=1}^{n}u(x_{i},10,100,4)\\ y_{i}=1+\frac{x_{i}+1}{4}\\ u(x_{i},a,k,m)=\left\{\begin{array}{l} k{\left(x_{i}-a\right)}^{m},x^{i}>a\\ 0,-a\leq x_{i}\leq a\\ k{\left(-x_{i}-z\right)}^{m},x_{i}<a \end{array}\right. \end{array}$	30	[−50, 50]	0
$\begin{array}{l} f_{13}(x)=0.1\left\{\sin^{2}\left(3\pi x_{1}+\sum_{i=1}^{n}\begin{array}{l} {\left(x_{i}-1\right)}^{2}[1+\sin^{2}(3\pi x_{i}+1)]\\ +{\left(x_{n}-1\right)}^{2}[1+\sin^{2}(2\pi x_{n})] \end{array}\right)\right\}\\ +\sum_{i=1}^{n}u(x_{i},5,100,4) \end{array}$	30	[−50, 50]	0
$f_{14}(x)={\left(\frac{1}{500}+\sum_{j=1}^{25}\frac{1}{j+\sum_{i=1}^{2}{\left(x_{i}-a_{ij}\right)}^{6}}\right)}^{-1}$	2	[−65, 65]	0.998
$f_{15}(x)=4x_{1}^{2}-2.1x_{1}^{4}+\frac{1}{3}x_{1}^{6}+x_{1}x_{2}-4x_{2}^{2}+4x_{2}^{4}$	2	[−5, 5]	−1.0316
$f_{16}(x)=-\frac{1+\cos(12\sqrt{x_{1}^{2}+x_{2}^{2}})}{0.5(x_{1}^{2}+x_{2}^{2})+2}$	2	[−5.12, 5.12]	−1
$\begin{array}{l} f_{17}(x)=[1+{\left(x_{1}+x_{2}+1\right)}^{2}\left(19-14x_{1}+3x_{1}^{2}-14x_{2}+6x_{1}x_{2}+3x_{2}^{2}\right)]\\ \times [30+{\left(2x_{1}-3x_{2}\right)}^{2}(18-32x_{1}+12x_{1}^{2}+48x_{2}-36x_{1}x_{2}+27x_{2}^{2})] \end{array}$	2	[−2, 2]	3
$f_{18}(x)=-\sum_{i=1}^{5}{\left[(x-a_{i}){\left(x-a_{i}\right)}^{T}+c_{i}\right]}^{-1}$	4	[0, 10]	−10.1532
$f_{19}(x)=-\sum_{i=1}^{7}{\left[(x-a_{i}){\left(x-a_{i}\right)}^{T}+c_{i}\right]}^{-1}$	4	[0, 10]	−10.4029
$f_{20}(x)=-\sum_{i=1}^{10}{\left[(x-a_{i}){\left(x-a_{i}\right)}^{T}+c_{i}\right]}^{-1}$	4	[0, 10]	−10.5364
$f_{21}(x)=-\cos(x_{1})\cos(x_{2})\exp(-{\left(x_{1}-\pi \right)}^{2}-{\left(x_{2}-\pi \right)}^{2})$	2	[−2π,2π]	−1
$f_{22}(x)=0.5+\frac{\sin^{2}\left(\sqrt{x_{1}^{2}+x_{2}^{2}}\right)-0.5}{{\left(1+0.001(x_{1}^{2}+x_{2}^{2})\right)}^{2}}$	2	[−100, 100]	−1
$f_{23}(x)=\sum_{i=1}^{n}x_{i}\sin(x_{i})+0.1x_{i}$	10	[−10, 10]	0

**Table 2:** Numerical statistics results of MSBKA and mainstream algorithms on benchmark functions.

Function	Result	HBO	MOA	CPO	DRA	SOO	EAO	HEOA	SFOA	TGCOA	H5N1	MShOA	BKA	MSBKA
$f_{1}$	Best	4.0 × 10^−132^	0	4.2 × 10^−259^	0	0	0	1.7 × 10^−190^	0	0	0	0	4.3 × 10^−220^	0
	Worst	2.4 × 10^−116^	0	8.7 × 10^−163^	0	0	0	4.9 × 10^−159^	0	0	0	0	5.5 × 10^−170^	0
	Mean	8.2 × 10^−118^	0	2.9 × 10^−164^	0	0	0	1.6 × 10^−160^	0	0	0	0	1.8 × 10^−171^	0
	Std	4.5 × 10^−117^	0	0	0	0	0	9.0 × 10^−160^	0	0	0	0	0	0
$f_{2}$	Best	4.20 × 10^−67^	3.7 × 10^−234^	6.6 × 10^−131^	0	0	0	7.51 × 10^−94^	0	0	0	0	4.3 × 10^−109^	0
	Worst	3.67 × 10^−59^	3.9 × 10^−224^	3.95 × 10^−88^	0	0	0	9.13 × 10^−71^	0	0	0	0	1.41 × 10^−86^	0
	Mean	2.16 × 10^−60^	1.6 × 10^−225^	1.34 × 10^−89^	0	0	0	3.04 × 10^−72^	0	0	0	0	5.01 × 10^−88^	0
	Std	8.04 × 10^−60^	0	7.21 × 10^−89^	0	0	0	1.67 × 10^−71^	0	0	0	0	2.58 × 10^−87^	0
$f_{3}$	Best	5.1 × 10^−136^	0	1.1 × 10^−298^	0	0	0	1.2 × 10^−216^	0	0	0	0	1.8 × 10^−219^	0
	Worst	8.8 × 10^−115^	2.1 × 10^−291^	2.3 × 10^−152^	0	0	0	3.3 × 10^−207^	0	0	0	0	4.1 × 10^−180^	0
	Mean	2.9 × 10^−116^	7.0 × 10^−293^	7.8 × 10^−154^	0	0	0	1.3 × 10^−208^	0	0	0	0	1.4 × 10^−181^	0
	Std	1.6 × 10^−115^	0	4.3 × 10^−153^	0	0	0	0	0	0	0	0	0	0
$f_{4}$	Best	4.93 × 10^−61^	1.9 × 10^−196^	5.7 × 10^−144^	0	0	0	1.12 × 10^−58^	0	0	0	0	6.5 × 10^−110^	0
	Worst	3.82 × 10^−6^	1.9 × 10^−187^	2.25 × 10^−81^	0	3.1 × 10^−298^	0	1.35 × 10^−48^	0	0	0	0	1.50 × 10^−96^	0
	Mean	1.27 × 10^−7^	6.5 × 10^−189^	7.50 × 10^−83^	0	1.1 × 10^−299^	0	5.06 × 10^−50^	0	0	0	0	5.02 × 10^−98^	0
	Std	6.97 × 10^−7^	0	4.11 × 10^−82^	0	0	0	2.45 × 10^−49^	0	0	0	0	2.74 × 10^−97^	0
$f_{5}$	Best	0.003200	21.76792	1.17 × 10^−5^	7.49 × 10^−9^	26.80666	28.99291	0.100026	26.58717	28.73990	23.7727	28.90589	24.66945	23.22636
	Worst	26.29904	25.86895	27.68064	9.25 × 10^−5^	28.92212	29.00000	3.355766	28.40617	28.91012	24.90451	28.99908	28.95534	28.71352
	Mean	19.09326	23.24124	22.11816	2.02 × 10^−5^	28.42710	28.99894	0.894984	27.35594	28.81234	24.40481	28.96989	26.37262	25.74011
	Std	11.28890	0.991052	10.06578	2.59 × 10^−5^	0.641270	0.001601	0.779963	0.404153	0.049787	0.29996	0.023669	1.304005	1.620269
$f_{6}$	Best	2.52 × 10^−6^	1.40 × 10^−10^	7.89 × 10^−6^	2.599605	6.40 × 10^−7^	6.822796	0.000704	0.018695	1.554598	5.33 × 10^−14^	6.004204	3.93 × 10^−5^	2.10 × 10^−12^
	Worst	0.003878	0.245149	0.531150	7.500000	0.144113	7.500000	0.913249	0.158590	5.451202	2.37 × 10^−12^	7.452951	5.236624	0.542759
	Mean	0.001059	0.008264	0.018478	6.771447	0.012847	7.444968	0.189272	0.063071	4.100679	6.98 × 10^−13^	6.876753	0.998784	0.018093
	Std	0.000728	0.044741	0.096859	1.280777	0.029868	0.126537	0.236452	0.040564	0.953789	6.07 × 10^−13^	0.427249	1.667562	0.099093
$f_{7}$	Best	2.33 × 10^−5^	1.33 × 10^−5^	1.48 × 10^−8^	4.85 × 10^−6^	1.55 × 10^−6^	2.17 × 10^−5^	4.24 × 10^−6^	8.04 × 10^−5^	5.87 × 10^−8^	2.37 × 10^−6^	5.65 × 10^−7^	1.20 × 10^−5^	4.45 × 10^−6^
	Worst	0.000507	0.000127	0.000195	0.000597	6.82 × 10^−5^	0.009797	0.000215	0.005138	8.05 × 10^−5^	0.000276	0.000190	0.000281	0.000153
	Mean	0.000180	5.83 × 10^−5^	5.04 × 10^−5^	0.000182	2.02 × 10^−5^	0.001100	6.13 × 10^−5^	0.00119	1.48 × 10^−5^	9.58 × 10^−5^	4.66 × 10^−5^	8.82 × 10^−5^	6.81 × 10^−5^
	Std	0.000112	3.04 × 10^−5^	5.09 × 10^−5^	0.000185	1.60 × 10^−5^	0.001797	5.77 × 10^−5^	0.001162	1.75 × 10^−5^	7.73 × 10^−5^	4.62 × 10^−5^	6.96 × 10^−5^	4.20 × 10^−5^
$f_{8}$	Best	−11,222.4	−9435.90	−8960.89	−12,566.1	−10,100.4	−5334.61	−8751.86	−9031.69	−6016.24	−12,569.5	−6528.30	−11,634.0	−17,002.5
	Worst	−7324.82	−7877.82	−1855.90	−8511.67	−8420.93	−3814.16	−5878.09	−5242.77	−3757.39	−12,332.6	−4150.61	−4906.21	−8947.67
	Mean	−10,140.0	−8616.58	−4387.13	−10,661.2	−9390.49	−4368.27	−7409.62	−6800.53	−4977.17	−12,478.7	−5160.04	−9117.53	−12,702.3
	Std	808.5363	404.5217	2537.775	1033.626	439.7259	315.9835	747.4668	902.1782	532.9380	91.65402	589.0267	1671.524	2010.001
$f_{9}$	Best	0	0	0	0	0	0	0	0	0	0	0	0	0
	Worst	26.17011	0	0	0	0	0	37.91576	0	0	0	0	0	0
	Mean	5.002825	0	0	0	0	0	29.55569	0	0	0	0	0	0
	Std	8.631902	0	0	0	0	0	5.895935	0	0	0	0	0	0
$f_{10}$	Best	4.44 × 10^−16^	4.44 × 10^−16^	4.44 × 10^−16^	4.44 × 10^−16^	4.44 × 10^−16^	4.44 × 10^−16^	4.44 × 10^−16^	4.44 × 10^−16^	4.44 × 10^−16^	4.44 × 10^−16^	4.44 × 10^−16^	4.44 × 10^−16^	4.44 × 10^−16^
	Worst	4.00 × 10^−15^	4.44 × 10^−16^	4.44 × 10^−16^	4.44 × 10^−16^	4.00 × 10^−15^	4.44 × 10^−16^	4.44 × 10^−16^	4.44 × 10^−16^	4.44 × 10^−16^	4.44 × 10^−16^	4.44 × 10^−16^	4.44 × 10^−16^	4.44 × 10^−16^
	Mean	2.46 × 10^−15^	4.44 × 10^−16^	4.44 × 10^−16^	4.44 × 10^−16^	2.34 × 10^−15^	4.44 × 10^−16^	4.44 × 10^−16^	4.44 × 10^−16^	4.44 × 10^−16^	4.44 × 10^−16^	4.44 × 10^−16^	4.44 × 10^−16^	4.44 × 10^−16^
	Std	1.79 × 10^−15^	0	0	0	1.80 × 10^−15^	0	0	0	0	0	0	0	0
$f_{11}$	Best	0	0	0	0	0	0	0	0	0	0	0	0	0
	Worst	0	0	0	0	0	0	0.080774	0	0	0	0	0	0
	Mean	0	0	0	0	0	0	0.029385	0	0	0	0	0	0
	Std	0	0	0	0	0	0	0.024916	0	0	0	0	0	0
$f_{12}$	Best	5.43 × 10^−6^	1.45 × 10^−11^	2.42 × 10^−11^	0.257206	4.23 × 10^−5^	1.217166	7.37 × 10^−5^	0.000541	0.208236	2.05 × 10^−15^	0.401642	2.54 × 10^−6^	2.59 × 10^−9^
	Worst	0.000205	0.103669	1.44 × 10^−7^	1.668971	1.205196	1.668971	0.855798	0.023898	0.965623	8.14 × 10^−13^	1.587615	0.736403	0.026854
	Mean	5.13 × 10^−5^	0.003458	2.41 × 10^−8^	1.291904	0.480386	1.609798	0.210297	0.002488	0.524968	5.16 × 10^−14^	1.155488	0.052429	0.003161
	Std	3.79 × 10^−5^	0.018927	2.92 × 10^−8^	0.381000	0.404680	0.119225	0.255890	0.004168	0.184857	1.48 × 10^−13^	0.294128	0.173112	0.006773
$f_{13}$	Best	2.00 × 10^−5^	8.96 × 10^−12^	1.18 × 10^−11^	8.36 × 10^−11^	0.086952	2.999258	4.77 × 10^−5^	0.025473	2.971278	2.06 × 10^−12^	2.810923	0.570024	1.13 × 10^−12^
	Worst	0.014970	0.141320	0.001909	2.71 × 10^−7^	2.704415	3.000000	0.013626	0.199407	2.989654	0.098883	2.999888	2.998022	0.010990
	Mean	0.001073	0.030498	6.41 × 10^−5^	6.46 × 10^−8^	2.244892	2.999858	0.001498	0.083601	2.979634	0.016383	2.987648	1.328206	0.001832
	Std	0.002657	0.042212	0.000349	7.60 × 10^−8^	0.647034	0.000212	0.002385	0.043775	0.005393	0.037262	0.037692	0.597340	0.004165
$f_{14}$	Best	0.998004	0.998004	0.998004	2.112136	0.998004	0.998004	0.998004	0.998004	0.998004	0.998004	0.998004	0.998004	0.998004
	Worst	0.998004	0.998004	12.67051	12.67051	2.982105	0.998119	12.67051	0.998004	12.67051	0.998004	12.67051	0.998004	0.998004
	Mean	0.998004	0.998004	9.628582	8.528383	1.130277	0.998013	8.873013	0.998004	9.311944	0.998004	4.255800	0.998004	0.998004
	Std	2.26 × 10^−16^	1.09 × 10^−16^	4.398218	3.338405	0.503383	2.22 × 10^−5^	4.798950	1.60 × 10^−16^	4.687439	2.44 × 10^−16^	4.521685	4.12 × 10^−17^	1.01 × 10^−16^
$f_{15}$	Best	−1.03163	−1.03163	−1.03163	−1.03091	−1.03163	−1.03163	−1.03163	−1.03163	−1.03163	−1.03163	−1.03163	−1.03163	−1.03163
	Worst	−1.03163	−1.03163	−1.01828	−0.96695	−1.03163	−1.02999	−1.03141	−1.03163	−1.03163	−1.03163	−1.03162	−1.03163	−1.03163
	Mean	−1.03163	−1.03163	−1.03055	−1.01670	−1.03163	−1.03132	−1.03158	−1.03163	−1.03163	−1.03163	−1.03163	−1.03163	−1.03163
	Std	5.45 × 10^−16^	6.05 × 10^−16^	0.002572	0.018270	6.78 × 10^−16^	0.000390	6.16 × 10^−5^	7.53 × 10^−13^	6.18 × 10^−16^	1.93 × 10^−8^	9.63 × 10^−7^	6.71 × 10^−16^	6.32 × 10^−16^
$f_{16}$	Best	−1	−1	−1	−1	−1	−1	−1	−1	−1	−1	−1	−1	−1
	Worst	−1	−1	−1	−1	−1	−1	−1	−1	−1	−1	−1	−1	−1
	Mean	−1	−1	−1	−1	−1	−1	−1	−1	−1	−1	−1	−1	−1
	Std	0	0	0	0	0	0	0	0	0	0	0	0	0
$f_{17}$	Best	3	3	3	3.931048	3	3.000314	3.000030	3	3	3	3	3	3
	Worst	3	3	3.000317	38.74167	3	3.011360	3.375273	3	30	3	3.000173	3	3
	Mean	3	3	3.000041	19.53724	3	3.002683	3.045402	3	3.9	3	3.000027	3	3
	Std	1.84 × 10^−15^	1.29 × 10^−15^	6.58 × 10^−5^	11.31279	1.32 × 10^−15^	0.002476	0.104753	1.38 × 10^−15^	4.929503	1.31 × 10^−15^	3.59 × 10^−5^	1.01 × 10^−15^	1.79 × 10^−15^
$f_{18}$	Best	−10.1532	−10.1532	−10.1532	−10.1532	−10.1532	−9.75430	−10.1454	−10.1532	−10.1532	−10.1532	−9.45139	−10.1532	−10.1532
	Worst	−5.05520	−5.10064	−10.1532	−10.1532	−4.84084	−4.67123	−6.92940	−10.1532	−2.61627	−5.0552	−4.74032	−10.1532	−2.63047
	Mean	−8.79373	−9.98475	−10.1532	−10.1532	−8.09275	−8.21814	−8.98634	−10.1532	−5.48654	−7.05924	−5.56236	−10.1532	−9.40093
	Std	2.292961	0.922461	1.21 × 10^−6^	3.85 × 10^−6^	2.448276	1.16749	0.990744	5.36 × 10^−12^	2.757751	2.340428	1.434193	5.52 × 10^−15^	2.295399
$f_{19}$	Best	−10.4029	−10.4029	−10.4029	−10.4028	−10.4029	−10.1920	−10.4015	−10.4029	−10.4029	−10.4029	−9.86446	−10.4029	−10.4029
	Worst	−2.76590	−10.4027	−10.4029	−10.4028	−4.90702	−6.53653	−6.97722	−10.4029	−2.45688	−5.08767	−4.81504	−3.72430	−3.72430
	Mean	−9.08532	−10.4029	−10.4029	−10.4028	−7.99926	−8.39715	−9.44603	−10.4029	−5.45487	−8.67301	−5.94171	−10.1803	−9.28983
	Std	2.461798	5.05 × 10^−5^	1.59 × 10^−6^	5.22 × 10^−6^	2.647310	1.110691	0.996354	1.33 × 10^−10^	2.932655	2.438102	1.670668	1.219347	2.531532
$f_{20}$	Best	−10.5364	−10.5364	−10.5364	−10.5363	−10.5364	−10.4923	−10.5362	−10.5364	−10.5364	−10.5364	−10.0904	−10.5364	−10.5364
	Worst	−3.83543	−10.5360	−10.5364	−10.5362	−4.94044	−5.61404	−5.12821	−10.5364	−2.80613	−5.12848	−4.43317	−10.5364	−3.83543
	Mean	−9.57564	−10.5364	−10.5364	−10.5363	−9.16810	−8.33815	−9.72042	−10.5364	−5.36153	−10.1171	−5.33110	−10.5364	−9.86631
	Std	2.153055	9.98 × 10^−5^	4.09 × 10^−6^	2.63 × 10^−5^	2.359324	1.231194	1.113618	3.81 × 10^−12^	2.246415	1.322932	1.222042	1.13 × 10^−14^	2.044661
$f_{21}$	Best	−1	−1	−1	−1	−1	−1	−1	−1	−1	−1	−1	−1	−1
	Worst	−1	−1	−1	−1	−1	−0.99833	−0.99992	−1	−1	−1	−0.99951	−1	−1
	Mean	−1	−1	−1	−1	−1	−0.99975	−0.99999	−1	−1	−1	−0.99993	−1	−1
	Std	0	0	6.21 × 10^−9^	1.24 × 10^−7^	0	0.000362	1.59 × 10^−5^	0	0	0	0.000113	0	0
$f_{22}$	Best	−1	−1	−1	−1	−1	−1	−1	−1	−1	−1	−1	−1	−1
	Worst	−1	−1	−1	−1	−1	−1	−0.99028	−1	−1	−1	−1	−1	−1
	Mean	−1	−1	−1	−1	−1	−1	−0.99805	−1	−1	−1	−1	−1	−1
	Std	0	0	0	0	0	0	0.003952	0	0	0	0	0	0
$f_{23}$	Best	5.86 × 10^−77^	6.1 × 10^−267^	3.1 × 10^−132^	0	0	0	2.10 × 10^−98^	0	0	0	0	2.7 × 10^−112^	0
	Worst	8.06 × 10^−8^	1.0 × 10^−257^	7.26 × 10^−83^	0	0	0	0.299463	0	0	0	0	1.62 × 10^−88^	0
	Mean	5.12 × 10^−9^	3.6 × 10^−259^	2.43 × 10^−84^	0	0	0	0.013477	0	0	0	0	5.41 × 10^−90^	0
	Std	1.53 × 10^−8^	0	1.33 × 10^−83^	0	0	0	0.057094	0	0	0	0	2.96 × 10^−89^	0

**Table 3:** Numerical results of MSBKA and mainstream algorithms in the Wilcoxon rank-sum test.

Function	HBO	MOA	CPO	DRA	SOO	EAO	MSO	SFOA	TGCOA	H5N1	MShOA	BKA
$f_{1}$	1.21 × 10^−12^	N/A	1.21 × 10^−12^	N/A	N/A	N/A	1.21 × 10^−12^	N/A	N/A	N/A	N/A	1.21 × 10^−12^
$f_{2}$	1.21 × 10^−12^	1.21 × 10^−12^	1.21 × 10^−12^	N/A	N/A	N/A	1.21 × 10^−12^	N/A	N/A	N/A	N/A	1.21 × 10^−12^
$f_{3}$	1.21 × 10^−12^	2.21 × 10^−6^	1.21 × 10^−12^	N/A	N/A	N/A	1.21 × 10^−12^	N/A	N/A	N/A	N/A	1.21 × 10^−12^
$f_{4}$	1.21 × 10^−12^	1.21 × 10^−12^	1.21 × 10^−12^	N/A	1.66 × 10^−11^	N/A	1.21 × 10^−12^	N/A	N/A	N/A	N/A	1.21 × 10^−12^
$f_{5}$	0.347828	4.18 × 10^−9^	0.085000	3.02 × 10^−11^	7.12 × 10^−9^	3.00 × 10^−11^	3.02 × 10^−11^	2.13 × 10^−5^	3.02 × 10^−11^	0.000770	3.02 × 10^−11^	0.031466
$f_{6}$	8.10 × 10^−10^	0.599689	6.72 × 10^−10^	3.02 × 10^−11^	5.46 × 10^−9^	2.98 × 10^−11^	4.62 × 10^−10^	5.57 × 10^−10^	3.02 × 10^−11^	3.34 × 10^−11^	3.02 × 10^−11^	2.87 × 10^−10^
$f_{7}$	2.96 × 10^−5^	0.355472	0.059428	0.037782	3.83 × 10^−6^	1.16 × 10^−7^	0.245814	1.07 × 10^−9^	4.80 × 10^−7^	0.318304	0.020681	0.446419
$f_{8}$	2.57 × 10^−7^	5.49 × 10^−11^	3.34 × 10^−11^	2.43 × 10^−5^	5.07 × 10^−10^	3.02 × 10^−11^	3.02 × 10^−11^	3.34 × 10^−11^	3.02 × 10^−11^	0.491783	3.02 × 10^−11^	2.39 × 10^−8^
$f_{9}$	0.002788	N/A	N/A	N/A	N/A	N/A	4.57 × 10^−12^	N/A	N/A	N/A	N/A	N/A
$f_{10}$	1.43 × 10^−6^	N/A	N/A	N/A	3.80 × 10^−6^	N/A	N/A	N/A	N/A	N/A	N/A	N/A
$f_{11}$	N/A	N/A	N/A	N/A	N/A	N/A	1.31 × 10^−7^	N/A	N/A	N/A	N/A	N/A
$f_{12}$	0.000399	0.003501	5.00 × 10^−9^	3.02 × 10^−11^	1.69 × 10^−9^	3.00 × 10^−11^	1.07 × 10^−9^	0.000284	3.02 × 10^−11^	3.02 × 10^−11^	3.02 × 10^−11^	7.74 × 10^−6^
$f_{13}$	6.74 × 10^−6^	0.002052	0.023243	1.87 × 10^−5^	3.02 × 10^−11^	2.92 × 10^−11^	6.74 × 10^−6^	3.02 × 10^−11^	3.02 × 10^−11^	7.20 × 10^−5^	3.02 × 10^−11^	3.02 × 10^−11^
$f_{14}$	0.004303	0.710179	3.13 × 10^−12^	3.16 × 10^−12^	0.722811	3.16 × 10^−12^	3.16 × 10^−12^	0.001410	1.39 × 10^−11^	0.000249	3.16 × 10^−12^	0.304902
$f_{15}$	0.001991	0.267806	7.57 × 10^−12^	7.57 × 10^−12^	0.005466	7.57 × 10^−12^	7.57 × 10^−12^	5.55 × 10^−6^	0.569127	0.503276	7.57 × 10^−12^	0.024637
$f_{16}$	N/A	N/A	N/A	N/A	N/A	N/A	N/A	N/A	N/A	N/A	N/A	N/A
$f_{17}$	0.007163	0.066462	2.17 × 10^−11^	2.17 × 10^−11^	0.127159	2.17 × 10^−11^	2.17 × 10^−11^	0.022229	0.007398	0.151530	2.17 × 10^−11^	0.840517
$f_{18}$	0.776364	0.396794	6.07 × 10^−8^	6.07 × 10^−8^	0.262031	6.07 × 10^−8^	6.07 × 10^−8^	1.58 × 10^−6^	7.54 × 10^−9^	1.62 × 10^−7^	6.07 × 10^−8^	0.282004
$f_{19}$	0.685096	0.559360	5.16 × 10^−6^	5.16 × 10^−6^	0.175046	5.16 × 10^−6^	5.16 × 10^−6^	7.71 × 10^−6^	1.20 × 10^−8^	0.001306	5.16 × 10^−6^	0.215899
$f_{20}$	0.269905	0.066540	1.17 × 10^−7^	9.16 × 10^−8^	0.018411	9.16 × 10^−8^	9.16 × 10^−8^	4.86 × 10^−6^	2.02 × 10^−8^	0.014383	9.16 × 10^−8^	0.242293
$f_{21}$	N/A	N/A	1.66 × 10^−11^	1.21 × 10^−12^	N/A	1.21 × 10^−12^	1.21 × 10^−12^	N/A	N/A	N/A	1.21 × 10^−12^	N/A
$f_{22}$	N/A	N/A	N/A	N/A	N/A	N/A	0.000656	N/A	N/A	N/A	N/A	N/A
$f_{23}$	1.21 × 10^−12^	1.21 × 10^−12^	1.21 × 10^−12^	N/A	N/A	N/A	1.21 × 10^−12^	N/A	N/A	N/A	N/A	1.21 × 10^−12^

**Table 4:** Numerical results of MSBKA, RBKA, SBKA, EBKA, and BKA on benchmark functions.

Function	Result	RBKA	SBKA	EBKA	BKA	MSBKA
$f_{1}$	Best	3.6 × 10^−219^	0	1.4 × 10^−237^	4.3 × 10^−220^	0
	Worst	2.8 × 10^−159^	1.1 × 10^−300^	3.4 × 10^−218^	5.5 × 10^−170^	0
	Mean	9.3 × 10^−161^	3.6 × 10^−302^	1.1 × 10^−219^	1.8 × 10^−171^	0
	Std	5.1 × 10^−160^	0	0	0	0
$f_{2}$	Best	4.0 × 10^−110^	6.1 × 10^−212^	3.6 × 10^−121^	4.3 × 10^−109^	0
	Worst	6.74 × 10^−92^	3.8 × 10^−141^	4.2 × 10^−111^	1.41 × 10^−86^	0
	Mean	2.25 × 10^−93^	1.3 × 10^−142^	1.6 × 10^−112^	5.01 × 10^−88^	0
	Std	1.23 × 10^−92^	7.0 × 10^−142^	7.7 × 10^−112^	2.58 × 10^−87^	0
$f_{3}$	Best	6.3 × 10^−220^	0	6.5 × 10^−227^	1.8 × 10^−219^	0
	Worst	5.5 × 10^−163^	0	7.6 × 10^−208^	4.1 × 10^−180^	0
	Mean	1.8 × 10^−164^	0	2.5 × 10^−209^	1.4 × 10^−181^	0
	Std	0	0	0	0	0
$f_{4}$	Best	1.3 × 10^−109^	2.2 × 10^−212^	2.9 × 10^−136^	6.5 × 10^−110^	0
	Worst	1.38 × 10^−82^	2.1 × 10^−151^	7.2 × 10^−122^	1.50 × 10^−96^	0
	Mean	4.61 × 10^−84^	6.9 × 10^−153^	2.5 × 10^−123^	5.02 × 10^−98^	0
	Std	2.52 × 10^−83^	3.8 × 10^−152^	1.3 × 10^−122^	2.74 × 10^−97^	0
$f_{5}$	Best	24.25563	22.10280	23.52517	24.66945	23.22636
	Worst	28.89462	28.83663	28.69909	28.95534	28.71352
	Mean	26.43481	24.77070	25.30940	26.37262	25.74011
	Std	1.247348	1.211486	1.271698	1.304005	1.620269
$f_{6}$	Best	2.90 × 10^−5^	7.21 × 10^−6^	4.50 × 10^−11^	3.93 × 10^−5^	2.10 × 10^−12^
	Worst	3.754495	0.757465	7.70 × 10^−5^	5.236624	0.542759
	Mean	0.349873	0.284266	2.29 × 10^−5^	0.998784	0.018093
	Std	0.870375	0.204645	3.06 × 10^−5^	1.667562	0.099093
$f_{7}$	Best	5.30 × 10^−6^	1.28 × 10^−5^	7.84 × 10^−6^	1.20 × 10^−5^	4.45 × 10^−6^
	Worst	0.000308	0.000271	0.000142	0.000281	0.000153
	Mean	8.43 × 10^−5^	9.05 × 10^−5^	4.91 × 10^−5^	8.82 × 10^−5^	6.81 × 10^−5^
	Std	7.60 × 10^−5^	6.58 × 10^−5^	4.04 × 10^−5^	6.96 × 10^−5^	4.20 × 10^−5^
$f_{8}$	Best	−11,306.6	−12,947.1	−11,733.7	−11,634.0	−17,002.5
	Worst	−5144.97	−6027.67	−7308.04	−4906.21	−8947.67
	Mean	−9316.77	−9420.36	−9497.69	−9117.53	−12,702.3
	Std	1344.450	1755.103	1161.631	1671.524	2010.001
$f_{9}$	Best	0	0	0	0	0
	Worst	0	0	0	0	0
	Mean	0	0	0	0	0
	Std	0	0	0	0	0
$f_{10}$	Best	4.44 × 10^−16^	4.44 × 10^−16^	4.44 × 10^−16^	4.44 × 10^−16^	4.44 × 10^−16^
	Worst	4.44 × 10^−16^	4.44 × 10^−16^	4.44 × 10^−16^	4.44 × 10^−16^	4.44 × 10^−16^
	Mean	4.44 × 10^−16^	4.44 × 10^−16^	4.44 × 10^−16^	4.44 × 10^−16^	4.44 × 10^−16^
	Std	0	0	0	0	0
$f_{11}$	Best	0	0	0	0	0
	Worst	0	0	0	0	0
	Mean	0	0	0	0	0
	Std	0	0	0	0	0
$f_{12}$	Best	2.02 × 10^−6^	0.006706	1.21 × 10^−10^	2.54 × 10^−6^	2.59 × 10^−9^
	Worst	0.665761	0.185280	0.010810	0.736403	0.026854
	Mean	0.046705	0.039401	0.001328	0.052429	0.003161
	Std	0.152983	0.046812	0.002950	0.173112	0.006773
$f_{13}$	Best	0.591135	0.154633	2.46 × 10^−9^	0.570024	1.13 × 10^−12^
	Worst	2.994766	2.993412	0.011028	2.998022	0.010990
	Mean	1.512737	1.035574	0.000386	1.328206	0.001832
	Std	0.763751	0.647302	0.002010	0.597340	0.004165
$f_{14}$	Best	0.998004	0.998004	0.998004	0.998004	0.998004
	Worst	1.992031	1.992031	4.950491	0.998004	0.998004
	Mean	1.031138	1.031138	1.129753	0.998004	0.998004
	Std	0.181484	0.181484	0.721622	4.12 × 10^−17^	1.01 × 10^−16^
$f_{15}$	Best	−1.03163	−1.03163	−1.03163	−1.03163	−1.03163
	Worst	−1.03163	−1.03163	−1.03163	−1.03163	−1.03163
	Mean	−1.03163	−1.03163	−1.03163	−1.03163	−1.03163
	Std	6.45 × 10^−16^	6.39 × 10^−16^	6.52 × 10^−16^	6.71 × 10^−16^	6.32 × 10^−16^
$f_{16}$	Best	−1	−1	−1	−1	−1
	Worst	−1	−1	−1	−1	−1
	Mean	−1	−1	−1	−1	−1
	Std	0	0	0	0	0
$f_{17}$	Best	3	3	3	3	3
	Worst	3	3	3	3	3
	Mean	3	3	3	3	3
	Std	7.09 × 10^−16^	6.55 × 10^−16^	1.42 × 10^−15^	1.01 × 10^−15^	1.79 × 10^−15^
$f_{18}$	Best	−10.1532	−10.1532	−10.1532	−10.1532	−10.1532
	Worst	−10.1532	−10.1532	−10.1532	−10.1532	−2.63047
	Mean	−10.1532	−10.1532	−10.1532	−10.1532	−9.40093
	Std	5.48 × 10^−15^	5.70 × 10^−15^	2.53 × 10^−14^	5.52 × 10^−15^	2.295399
$f_{19}$	Best	−10.4029	−10.4029	−10.4029	−10.4029	−10.4029
	Worst	−3.72430	−2.76590	−2.76590	−3.72430	−3.72430
	Mean	−10.1803	−9.41661	−10.1484	−10.1803	−9.28983
	Std	1.219347	2.562275	1.394327	1.219347	2.531532
$f_{20}$	Best	−10.5364	−10.5364	−10.5364	−10.5364	−10.5364
	Worst	−10.5364	−2.42173	−10.5364	−10.5364	−3.83543
	Mean	−10.5364	−9.72513	−10.5364	−10.5364	−9.86631
	Std	1.78 × 10^−15^	2.475450	3.18 × 10^−15^	1.13 × 10^−14^	2.044661
$f_{21}$	Best	−1	−1	−1	−1	−1
	Worst	−1	−1	−1	−1	−1
	Mean	−1	−1	−1	−1	−1
	Std	0	0	0	0	0
$f_{22}$	Best	−1	−1	−1	−1	−1
	Worst	−1	−1	−1	−1	−1
	Mean	−1	−1	−1	−1	−1
	Std	0	0	0	0	0
$f_{23}$	Best	5.6 × 10^−113^	1.6 × 10^−212^	4.2 × 10^−122^	2.7 × 10^−112^	0
	Worst	1.62 × 10^−82^	1.7 × 10^−138^	2.4 × 10^−107^	1.62 × 10^−88^	0
	Mean	5.39 × 10^−84^	5.5 × 10^−140^	8.4 × 10^−109^	5.41 × 10^−90^	0
	Std	2.95 × 10^−83^	3.0 × 10^−139^	4.5 × 10^−108^	2.96 × 10^−89^	0

**Table 5:** Numerical results of MSBKA, RBKA, SBKA, EBKA, and BKA in the Wilcoxon rank-sum test.

Function	RBKA	SBKA	EBKA	BKA
$f_{1}$	1.21 × 10^−12^	0.333711	1.21 × 10^−12^	1.21 × 10^−12^
$f_{2}$	1.21 × 10^−12^	1.21 × 10^−12^	1.21 × 10^−12^	1.21 × 10^−12^
$f_{3}$	1.21 × 10^−12^	N/A	1.21 × 10^−12^	1.21 × 10^−12^
$f_{4}$	1.21 × 10^−12^	1.21 × 10^−12^	1.21 × 10^−12^	1.21 × 10^−12^
$f_{5}$	0.010763	0.019883	0.195791	0.031466
$f_{6}$	4.20 × 10^−10^	6.12 × 10^−10^	0.403538	2.87 × 10^−10^
$f_{7}$	0.818746	0.277189	0.074827	0.446419
$f_{8}$	6.52 × 10^−9^	2.20 × 10^−7^	1.31 × 10^−8^	2.39 × 10^−8^
$f_{9}$	N/A	N/A	N/A	N/A
$f_{10}$	N/A	N/A	N/A	N/A
$f_{11}$	N/A	N/A	N/A	N/A
$f_{12}$	1.53 × 10^−5^	3.20 × 10^−9^	0.982307	7.74 × 10^−6^
$f_{13}$	3.02 × 10^−11^	3.02 × 10^−11^	0.077272	3.02 × 10^−11^
$f_{14}$	0.677068	0.110894	0.677068	0.304902
$f_{15}$	0.529069	0.763881	0.326335	0.024637
$f_{16}$	N/A	N/A	N/A	N/A
$f_{17}$	N/A	0.768636	0.454528	0.840517
$f_{18}$	0.189185	0.087771	0.402213	0.282004
$f_{19}$	0.534780	0.883950	0.142172	0.215899
$f_{20}$	0.162188	0.645996	0.749628	0.242293
$f_{21}$	N/A	N/A	N/A	N/A
$f_{22}$	N/A	N/A	N/A	N/A
$f_{23}$	1.21 × 10^−12^	1.21 × 10^−12^	1.21 × 10^−12^	1.21 × 10^−12^

**Table 6:** Numerical statistics results of the three-bar truss.

Algorithm	Optimum Moderating Elements		Optimum Counterweight
	$A_{1}$	$A_{2}$	
BKA [14]	0.788675	0.408248	263.895843
SHO [14]	0.788898	0.40762	263.895881
TTAO [44]	0.788688	0.408213	263.8958431
MTBO [45]	0.78868	0.40825	263.8958434
AEFA [46]	0.78848449	0.408787881	263.9195433
PSA [46]	0.789351215	0.406573046	263.8958824
SCHO [47]	0.7886642	0.40827926	263.8958476
INFO [48]	0.788672734	0.408255081	263.8958434
APO [49]	0.7887	0.4082	263.89584338
BSLO [39]	0.78867930	0.40823651	263.8958434
FOX [39]	0.78870269	0.4081704	263.8958523
ARSCA [5]	0.7887	0.4081	263.8958
CPO [5]	0.7885	0.4088	263.8959
HEOA [9]	0.789	0.409	264
PKO [50]	0.7886870838	0.4082144942	263.8958435
SBOA [38]	0.789	0.409	264
MadDE [38]	0.789	0.408	264
SO [38]	0.789	0.407	264
RIME [38]	0.734	0.590	265
SFOA [10]	0.78868	0.40825	263.89584
MSBKA	0.765579	0.468012	263.8745

**Table 7:** Numerical statistics results of the tension/compression spring.

Algorithm	Optimum Moderating Elements			Optimum Counterweight
	d	D	P	
BKA [14]	0.051173	0.344426	12.047782	0.01267027
SHO [14]	0.0508	0.334800	11.702	0.012681
NRBO [51]	0.0517	0.3575	11.2382	0.0127
RKO [51]	0.0523	0.3717	10.4586	0.0127
TTAO [44]	0.051674	0.051674	11.31044	0.012665
WO [40]	0.05	0.3115	14.8923	0.012665
RCGO [52]	0.052866	0.385549	9.787463	0.012701
AGWO [52]	0.051082	0.34226	12.19919	0.012681
WaOA [53]	0.0519693	0.363467	10.9084	0.012672
INFO [48]	0.051555	0.353499	11.48034	0.012666
APO [49]	0.0517	0.3567	11.2890	0.01266523
BSLO [39]	0.051669	0.356226	11.31788	0.0126652
FOX [39]	0.051983	0.363808	10.88657	0.0126686
EHO [54]	0.051746	0.358097	11.208557	0.012665
LFD [55]	0.0517	0.3575	11.2442	0.0127
BBOA [55]	0.051344	0.334881	12.6223	0.012667
GRO [55]	0.0517082206	0.35717883	11.2619852	0.012665
MadDE [38]	0.0518	0.358	11.2	0.0127
RIME [38]	0.0693	0.940	2	0.0181
SBOA [38]	0.0517	0.357	11.3	0.0127
MSBKA	0.051785	0.353691	11.143834	0.012478

**Table 8:** Numerical statistics results of the gear train.

Algorithm	Optimum Moderating Elements				Optimum Cost-Effectiveness
	$n_{A}$	$n_{B}$	$n_{C}$	$n_{D}$	
CSA [56]	43	16	19	49	2.701 × 10^−12^
KOA [57]	44	20	16	50	2.700857 × 10^−12^
FLA [57]	44	16	20	49	2.700857 × 10^−12^
COA [57]	23	14	12	48	9.92158 × 10^−10^
RUN [57]	44	17	19	49	2.700857 × 10^−12^
SMA [57]	52	30	13	53	2.307816 × 10^−11^
DO [57]	49	16	19	44	2.700857 × 10^−12^
POA [57]	44	17	19	49	2.700857 × 10^−12^
PDO [58]	48	17	22	54	2.70 × 10^−12^
DMOA [58]	49	19	16	43	2.70 × 10^−12^
AOA [58]	49	19	19	54	2.70 × 10^−12^
SSA [58]	49	19	19	49	2.70 × 10^−12^
IEHO [59]	19	16	43	49	2.70085 × 10^−12^
ARO [60]	49	19	16	43	2.7009 × 10^−12^
GMO [61]	43	19	16	49	2.700857 × 10^−12^
GBO [44]	53	13	20	34	2.3078 × 10^−11^
TTAO [44]	43	16	19	49	2.70 × 10^−12^
WO [40]	43	16	19	43	2.700857 × 10^−12^
GCRA [62]	55	16	16	43	2.70 × 10^−12^
GOA [62]	49	19	16	43	2.70 × 10^−12^
MSBKA	56	18	20	45	1.7869 × 10^−19^

**Table 9:** Numerical statistics results of the car side impact.

Algorithm	Optimum Moderating Elements						Optimum Counterweight
	$x_{1}$	$x_{2}$	$x_{3}$	$x_{4}$	$x_{5}$	$x_{6}$	
	$x_{7}$	$x_{8}$	$x_{9}$	$x_{10}$	$x_{11}$		
WOA [63]	0.5	1.108001	0.534477	1.30577	0.5	1.473844	
	0.5	0.345	0.192	−19.69924	3.4816923		23.04216220
CSS [63]	0.5	1.184389	0.5	1.230036	0.5	1.5	
	0.5	0.280792	0.342425	−7.394733	0.042206		23.00733588
CLPSO [64]	0.5061	1.17379	0.5013	1.24706	0.5037	1.4956	
	0.5	0.345	0.345	−9.5985	3.3627		23.06244
BOA [64]	0.8246	1.03224	0.54007	1.35639	0.6377	1.26889	
	0.5854	0.192	0.345	−5.7333	0.4352		25.06573
HGSO [64]	0.5	1.22375	0.5	1.27111	0.5	1.31085	
	0.5	0.345	0.345	−4.3235	2.93676		23.43457
DOA [41]	0.5081	1.2021	0.5318	1.3052	0.5719	1.4954	
	0.5557	0.303	0.2585	−24.8171	3.4047		23.9682
DCS [41]	0.5772	1.2586	0.5195	1.2002	0.5463	1.258	
	0.5073	0.278	0.2669	2.0888	5.4035		23.9995
COA [41]	0.5	1.2791	0.5	1.2739	1.2828	0.5	
	0.5	0.2954	0.192	3.557	19.0792		25.2083
MSA [41]	0.5151	1.2684	0.5545	1.3737	0.5261	1.3484	
	0.7156	0.2869	0.2167	−7.2394	11.7869		25.2334
HLOA [41]	0.5	1.0669	0.8016	1.0704	0.504	1.4873	
	0.5	0.192	0.192	−29.9786	3.2119		23.6956
SCA [41]	0.5	1.2499	0.5	1.4521	0.5001	1.4946	
	0.5	0.345	0.192	−14.7797	−1.5647		24.3349
AROA [41]	0.5	1.5	0.5	1.2928	0.5	0.5	
	0.5	0.192	0.3195	8.8265	23.0874		25.3642
ETO [65]	0.50282	1.2414	0.51604	1.2201	0.60334	1.3878	
	0.5	0.74832	0.06747	2.2526	−7.2818		23.2574
SCHO [65]	0.5	1.10286	0.87088	0.88643	0.52609	1.49992	
	0.5	0.03508	0.19439	−30	−0.5913		23.7209
AOA [65]	0.5	1.2279	0.5	1.4332	0.5	1.5	
	0.5	0.61018	0.21619	0.00126	−0.0765		24.1125
HGS [65]	0.5	1.10612	1.11044	0.5	0.5	1.5	
	0.5	4.4 × 10^−9^	0.00000	−30	−6.0 × 10^−9^		23.8188
GJO [65]	0.5	1.20309	0.50327	1.28778	0.51053	1.5	
	0.5	0.00000	9.5 × 10^−5^	−22.115	−0.0536		23.4052
SCSO [66]	0.502366774	1.23533939	0.5	1.223008761	0.515267967	1.39187245	
	0.50003369	0.340647775	0.211950171	1.374158706	−7.77399175		23.35787723
SOA [66]	0.500139239	1.254868587	0.5	1.205871077	0.739233716	0.772309974	
	0.5	0.316999014	0.30308334	0.749660043	2.039711514		23.8070425
SFOA [10]	0.5	1.234	0.5	1.187	0.875	0.892	
	0.4	0.345	0.192	1.5	0.572		23.5616
MSBKA	0.5	1.11674	0.5	1.31276	0.5	1.5	
	0.5	0.34992	0.191	−19.53974	−0.02683		22.88942

**Table 10:** Numerical statistics results of the multiple-disk clutch brake.

Algorithm	Optimum Moderating Elements					Optimum Counterweight
	$r_{i}$	$r_{0}$	t	F	Z	
TLBO [67]	70	90	1	810	3	0.313657
MFO [68]	70	90	1	910	3	0.313656
MVO [69]	70	90	1	910	3	0.313656
CMVO [69]	70	90	1	910	3	0.313656
WCA [70]	70	90	1	910	3	0.313656
PVS [71]	70	90	1	980	3	0.31366
APSO [72]	76	96	1	840	3	0.337181
FSO [73]	70	90	1	870	3	0.31365661053
GOA [74]	71	92	1	835	3	0.3355146
GSA [75]	72	92	2	815	3	0.3175771
AEO [75]	70	90	1	810	3	0.3136566
AHA [76]	70	90	1	840	3	0.3136566
HBO [77]	70	90	1	1000	3	0.3136566
HGS [78]	70	90	1	1000	3	0.313657
MRFO [79]	70	90	1	835	3	0.3136566
GA [79]	72	92	1	918	3	0.321498
DE [79]	71	92	1	835	3	0.3355146
EPO [42]	70	90	1.5	1000	3	0.4704
RSO [42]	70	90	1	810	3	0.313657
MSBKA	70	90	1	987	2	0.27642

**Table 11:** Numerical statistics results of the rolling element bearing.

Algorithm	Optimum Moderating Elements					Optimum Cost-Effectiveness
	$D_{m}$	$D_{b}$	Z	$f_{i}$	$f_{o}$	
	$K_{D\min}$	$K_{D\max}$	ε	e	ζ	
PVS [71]	125.71906	21.42559	11	0.515	0.515	
	0.40043	0.68016	0.3	0.07999	0.7	81,859.74121
TLBO [67]	125.7191	21.42559	11	0.515	0.515	
	0.424266	0.633948	0.3	0.068858	0.799498	81,859.74
CPA [80]	125.722718	21.423301	11.001159	0.515	0.515	
	0.473508	0.617554	0.3	0.086504	0.680706	81,849.21039788
MFO [81]	125	21.032	10.965	0.515	0.515	
	0.5	0.675	0.3	0.02	0.61	84,002.524
GSA [81]	125	20.854	11.149	0.515	0.517	
	0.5	0.618	0.3	0.02	0.624	82,276.941
HS [81]	125	20.871	11.166	0.515	0.516	
	0.5	0.619	0.3	0.05	0.614	81,569.527
MVO [81]	125	21.322	10.973	0.515	0.515	
	0.5	0.687	0.3	0.03	0.61	84,491.266
SCA [81]	125	21.148	10.969	0.515	0.515	
	0.5	0.7	0.3	0.02	0.629	83,431.117
HHO [82]	125	21	11.09207	0.515	0.515	
	0.4	0.6	0.3	0.050474	0.6	83,011.88
RSA [82]	125.1722	21.29734	10.88521	0.515253	0.517764	
	0.41245	0.632338	0.301911	0.024395	0.6024	83,486.64
DE [83]	125	20.87123	11.16697	0.515	0.516	
	0.5	0.61951	0.301128	0.05024	0.614531	81,569.527
SSA [72]	125	20.77562	11.01247	0.515	0.515	
	0.5	0.61397	0.3	0.05004	0.61001	82,773.982
HPO [84]	125	21.875	10.777	0.515	0.515	
	0.4	0.7	0.3	0.029	0.6	83,918.4925
CS [79]	125.442787	21.205159	11	0.515	0.5416852	
	0.5	0.7	0.3	0.0975781	0.6015492	83,988.259
RUN [85]	125.2142	21.59796	11.4024	0.515	0.515	
	0.40059	0.61467	0.3053	0.02	0.63665	83,680.47
MGA [86]	125.718	21.8745119	10.7770658	0.51500082	0.51500299	
	0.405908353	0.65558802	0.30000415	0.07754492	0.6	83,912.87983
CGO [86]	125	21.875	10.777009	0.515	0.515	
	0.4	0.64620052	0.3	0.050152445	0.6	83,918.49253
EVO [86]	125.7190556	21.4255902	10.6955328	0.515	0.515	
	0.463182936	0.6999265	0.3	0.063431519	0.604213108	81,859.7415974
SELO [43]	126.3521	21.0299	11	0.515	0.515	
	0.4	0.6011	0.3	0.1	0.6004	83,805.29
LFD [43]	126.3999	21	11	0.515	0.5251	
	0.5	0.6	0.3	0.1	0.6	83,670.78
MSBKA	125	21.7648	11.3921	0.5151	0.5151	
	0.4	0.7	0.3	0.02	0.6	85,012.5432

## Data Availability

The data presented in this study are available on request from the corresponding author. The MATLAB R2022b code developed for this study is available from the corresponding author upon reasonable request.
